# MSGRL: A Motif-Driven Self-Supervised Graph Representation Learning Framework for Interpretable Molecular Property Prediction

**DOI:** 10.3390/molecules31173008

**Published:** 2026-08-27

**Authors:** You Wu, Yuxin Jiang, Xiaoyun Qi, Haitao Fu, Qiyu Tang, Wen Wang, Cheng Zeng, Guosheng Zhu

**Affiliations:** 1 School of Artificial Intelligence, Hubei University, Wuhan 430062, China; wuyou079@webmail.hzau.edu.cn (Y.W.); jyx@stu.hubu.edu.cn (Y.J.); fuhaitao@hubu.edu.cn (H.F.); 2 School of Computer Science, Hubei University, Wuhan 430062, China; 3 College of Informatics, Huazhong Agricultural University, Wuhan 430070, China; 4 School of Chemistry and Chemical Engineering, Hubei University, Wuhan 430062, China; qixiaoyun@hubu.edu.cn; 5 Key Laboratory of Intelligent Sensing System and Security (Hubei University), Ministry of Education, Wuhan 430062, China; 6 The Roslin Institute, University of Edinburgh, Edinburgh EH25 9RG, UK; tqyvet@gmail.com; 7 Institute for Neuroscience and Cardiovascular Research, Centre for Cardiovascular Science, University of Edinburgh, Edinburgh EH16 4TJ, UK

**Keywords:** molecular property prediction, molecular fragments, self-supervised learning, graph neural networks, variational graph autoencoder

## Abstract

Molecular property prediction is a fundamental task in drug discovery and chemical biology, where effective molecular representations are essential for accurate prediction. Learning transferable motif-level representations remains challenging because explicit motif annotations are scarce and existing representations may be altered during downstream supervised optimization. In this study, we propose MSGRL, a motif-driven self-supervised graph representation learning framework for interpretable molecular property prediction. MSGRL represents each molecule through a hierarchical graph structure, consisting of a motif-based graph for inter-motif organization and motif-specific atom-based graphs for intra-motif atomic structure. Its central design is to decouple label-agnostic intra-motif structural learning from label-dependent inter-motif property learning. An MPNN-GRU encoder is pretrained on motif-specific atom-based graphs using a variational motif graph autoencoder (VMGAE), which reconstructs the internal bond topology of motifs in a self-supervised manner. After pretraining, the intra-motif encoder is kept frozen, while the downstream module pools atom-level feature matrices into motif vectors, propagates information over the motif-based graph, and applies attention-based pooling for molecular property prediction. This design keeps the pretrained intra-motif representations fixed while allowing the inter-motif network and prediction head to adapt to individual downstream tasks. Experiments on eight MoleculeNet benchmark datasets show that MSGRL achieves the highest ROC-AUC scores on all five classification datasets and the lowest RMSE on Lipophilicity, while its performance on ESOL and FreeSolv is more mixed. Ablation studies further support the contributions of encoder freezing, motif-based graph construction, and attention-based pooling. Motif-level attribution analyses provide qualitative and dataset-level evidence regarding the substructures emphasized by the model. These results demonstrate the effectiveness of the proposed hierarchical representation strategy, particularly for the evaluated molecular classification tasks.

## 1. Introduction

Molecular property prediction is a critical task in drug discovery, pharmacology, and chemical biology, and accurate computational models can substantially reduce the cost and time associated with experimental screening [1,2,3]. Graph neural networks (GNNs) are widely used for this purpose because molecules can be naturally represented as graphs, with atoms as nodes and chemical bonds as edges [4,5]. Graph convolutional networks (GCNs) [4], message passing neural networks (MPNNs) [6], graph isomorphism networks (GINs) [5], AttentiveFP [7], GPS [8], and DeeperGCN [9] have progressively improved molecular representation learning through expressive message passing, attention, and global-context modeling.

Despite these advances, conventional atom-level GNNs primarily model local atom–bond connectivity and may not explicitly capture the hierarchical organization of molecules. Chemically relevant information can arise at multiple structural levels, including functional groups, recurring substructures, motifs, and molecular scaffolds. Accordingly, recent studies have incorporated semantic information [10], molecular tokenization [11], domain-informed chemical signals [12], geometric descriptors [13], conformational information [14], and three-dimensional molecular pretraining [15]. Haghighatlari et al. [16] further emphasized that successful chemical machine learning depends on the interplay among feature representation, data, and learning algorithms. These observations motivate molecular representations that capture chemically relevant structure beyond conventional atom-level connectivity.

Motif-based modeling provides one such strategy by organizing atoms into submolecular units and explicitly representing their relationships. This use of motifs should be distinguished from fragment-based drug discovery (FBDD), where small fragments primarily serve as starting points for compound identification and optimization through growing, merging, or linking strategies [17]. In MSGRL, BRICS-derived substructures are instead treated as hierarchical representation units. Each substructure retains its internal atom–bond topology, while relationships among substructures are represented through a higher-level graph. Thus, the proposed approach does not assume that motifs are intrinsically superior to fragments; its distinction lies in how the resulting substructures are represented and learned.

Existing motif- and fragment-aware methods have explored related hierarchical representations from different perspectives. HimGNN [18] jointly models atom- and motif-level representations, whereas FraGAT [19] integrates atom-, fragment-, and molecular-level information through multi-scale graph attention. Among self-supervised approaches, MGSSL [20] combines atom-level objectives with motif-generation tasks, HiMol [21] learns node–motif–graph representations through multi-level self-supervision, and MICRO-Graph [22] uses learned motifs to guide graph-to-subgraph contrastive learning. More recently, Long et al. [23] represented BRICS-derived motifs using pretrained MoLFormer embeddings and modeled their interactions with a GATv2-based graph network for drug-induced QT prolongation. Together, these studies demonstrate the value of substructure-aware molecular modeling, but they focus primarily on supervised hierarchical integration, motif-aware pretraining, contrastive learning, or task-specific interactions among pre-encoded motifs.

A remaining question is how to learn the internal structure of individual motifs independently of downstream property labels while retaining the flexibility to learn task-specific relationships among motifs. In pretraining-based methods such as MGSSL [20] and HiMol [21], pretrained molecular encoders are subsequently adapted during downstream fine-tuning. By contrast, explicitly separating label-agnostic intra-motif structural learning from label-dependent inter-motif property learning remains less explored. Such a separation keeps the pretrained intra-motif encoder independent of downstream gradient updates while allowing the inter-motif network to adapt to individual tasks.

To address this problem, we propose MSGRL, a Motif-driven Self-supervised Graph Representation Learning framework for interpretable molecular property prediction. The central contribution of MSGRL is a decoupled hierarchical learning strategy that separates intra-motif structural representation learning from downstream inter-motif property learning. Each BRICS-derived motif is retained as an atom-based graph, and an MPNN-GRU encoder is pretrained using a variational motif graph autoencoder (VMGAE) that reconstructs its internal bond topology. The pretrained intra-motif encoder is then frozen, while the downstream module learns task-specific relationships over the motif graph and aggregates motif representations through attention-based pooling. This design preserves self-supervised intra-motif representations while allowing the inter-motif network and prediction head to adapt to individual molecular property tasks.

We evaluate MSGRL on eight MoleculeNet benchmark datasets [24] covering classification and regression tasks. MSGRL achieves the highest ROC-AUC scores on all five evaluated classification datasets and the lowest RMSE on Lipophilicity, while its performance on ESOL and FreeSolv is more mixed. Ablation studies further examine the effects of encoder freezing, motif-based graph construction, and attention-based pooling. Motif-level attribution analyses provide additional qualitative and dataset-level evidence regarding the substructures emphasized by the model.

## 2. Results

### 2.1. Overall Framework

**Figure 1:**
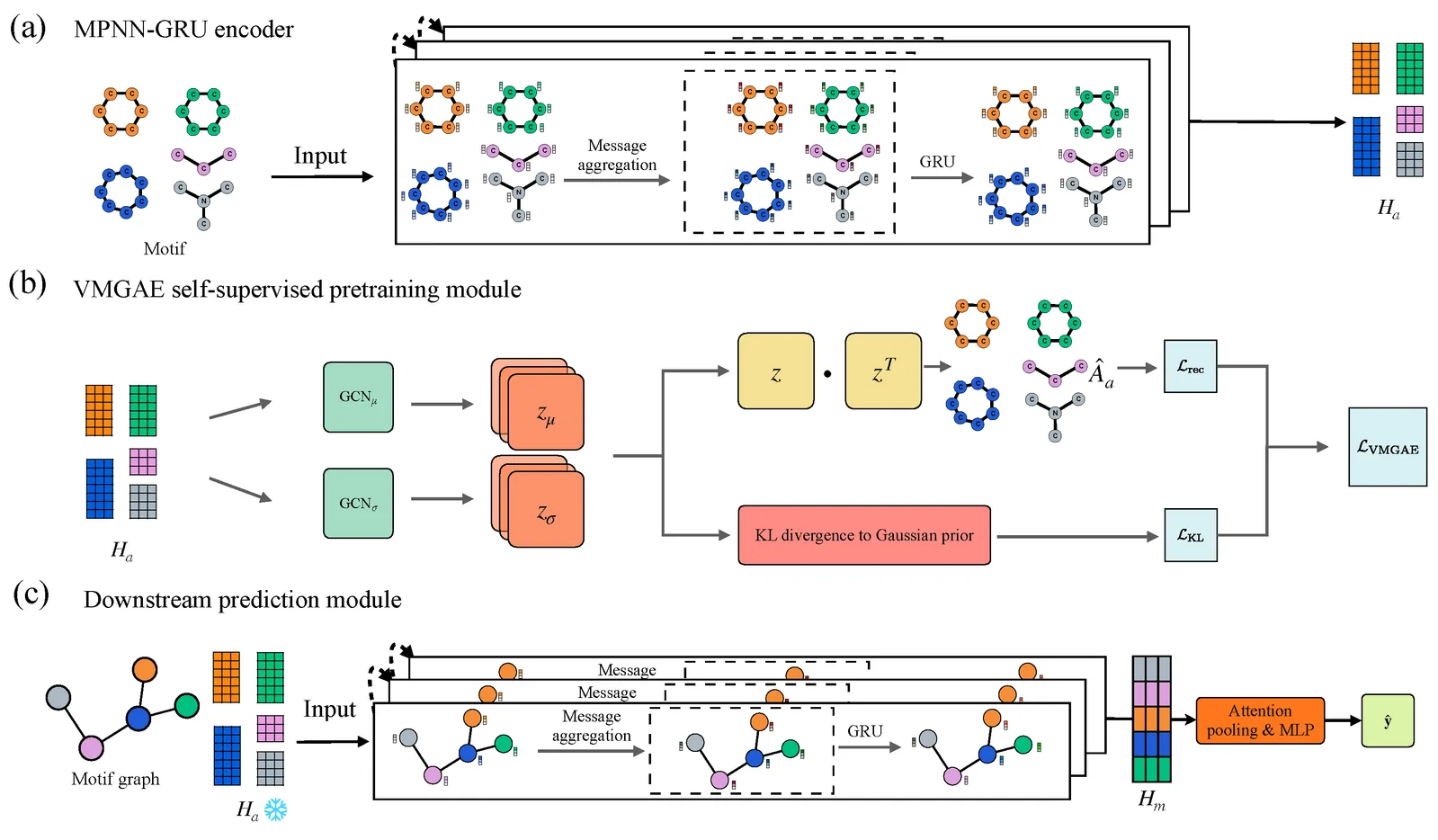
Overview of the MSGRL framework. (**a**) The MPNN-GRU encoder performs message passing and gated updates on motif-specific atom-based graphs to learn atom-level representations and produce an atom-level feature matrix for each motif. (**b**) During self-supervised pretraining, the VMGAE maps these representations into a variational latent space and reconstructs the intra-motif bond topology using an adjacency reconstruction objective together with KL divergence regularization. (**c**) During downstream prediction, the pretrained intra-motif encoder is kept frozen; atom-level feature matrices are pooled into motif vectors, propagated over the inter-motif graph, and aggregated by attention pooling before the task-specific MLP produces classification or regression outputs. The attention weights additionally provide motif-level importance cues for interpretation.

As illustrated in Figure 1, MSGRL consists of three main components: an MPNN-GRU encoder, a VMGAE self-supervised pretraining module, and a downstream prediction module. Specifically, each molecule is first represented as a motif-based graph in which motif nodes are connected according to their shared atoms. Each motif is further represented as an atom-based graph that describes its internal atomic structure. The MPNN-GRU encoder operates on the atom-based graphs of individual motifs to learn atom-level representations, which form a motif-level feature matrix for each motif. The VMGAE module further optimizes these atom-level and motif-level representations through a variational graph autoencoding objective in a self-supervised manner. Finally, the downstream prediction module uses the frozen pretrained encoder to obtain motif-level feature matrices. Each matrix is first pooled into a motif vector. The resulting motif vectors are then propagated over the motif-based graph and aggregated through attention-based pooling to obtain a molecular-level representation.

### 2.2. Performance Comparison with Baseline Models

We compare MSGRL with representative molecular graph learning models, including general graph architectures (GCN-MPNN [4], GIN-MPNN [5], AttentiveFP [7], GPS [8], DeeperGCN [9], VGAE [25], and MetaGIN [26]), motif- and fragment-aware methods (MICRO-Graph [22], MGSSL [20], HiMol [21], HimGNN [18], and FraGAT [19]), and geometry- or semantic-aware models (GemNet [27] and SCI [10]).

As shown in Table 1, MSGRL achieves the highest mean ROC-AUC on all five classification datasets and the lowest RMSE on Lipophilicity under the evaluated protocol. Two-sided Welch’s *t*-tests with Holm correction show that MSGRL significantly outperforms the strongest competing baseline on BACE, BBBP, SIDER, Tox21, and Lipophilicity ($p_{\mathrm{adj}}<0.05$; Supplementary Table S2). The difference on ClinTox is not statistically significant, whereas the strongest competing baseline significantly outperforms MSGRL on ESOL and FreeSolv.

Among the general-purpose GNN baselines, DeeperGCN and GPS show competitive performance on several datasets. However, these methods mainly operate on conventional molecular graph representations and do not explicitly incorporate motif-level inductive biases. For molecular property prediction, motif-level information can provide complementary structural priors beyond conventional atom-level connectivity.

Several motif- and subgraph-aware methods also show strong performance across the evaluated datasets. For example, FraGAT performs particularly well on BACE, BBBP, ClinTox, and Tox21, while HimGNN and HiMol show competitive results on several classification and regression benchmarks. MSGRL differs from these approaches by applying self-supervised pretraining to motif-specific atom-based graphs and keeping the pretrained intra-motif encoder fixed during downstream training. The motif-level predictor remains trainable and adapts to individual tasks.

MSGRL shows particularly strong performance on the classification benchmarks. For example, it achieves ROC-AUC values of 0.943 on SIDER and 0.947 on Tox21, outperforming the strongest competing baselines under the evaluated protocol. These results support the effectiveness of MSGRL for the evaluated multi-task classification settings. However, the present experiments do not isolate which specific dataset characteristics account for these improvements.

For regression tasks, MSGRL achieves the lowest RMSE of 0.438 on Lipophilicity, while its performance on ESOL and FreeSolv is more mixed. To complement the average RMSE values, we further report the maximum absolute prediction error for each regression dataset in Supplementary Table S1 and present predicted-versus-ground-truth scatter plots and distribution comparisons in Supplementary Figure S1. These analyses provide additional information on the largest observed prediction deviations and the agreement between predicted and experimental property distributions across the evaluated ranges. We note that this analysis does not constitute a formal activity-cliff evaluation, which would require explicitly identifying structurally similar molecular pairs with large property differences.

### 2.3. Ablation Study

**Figure 2:**
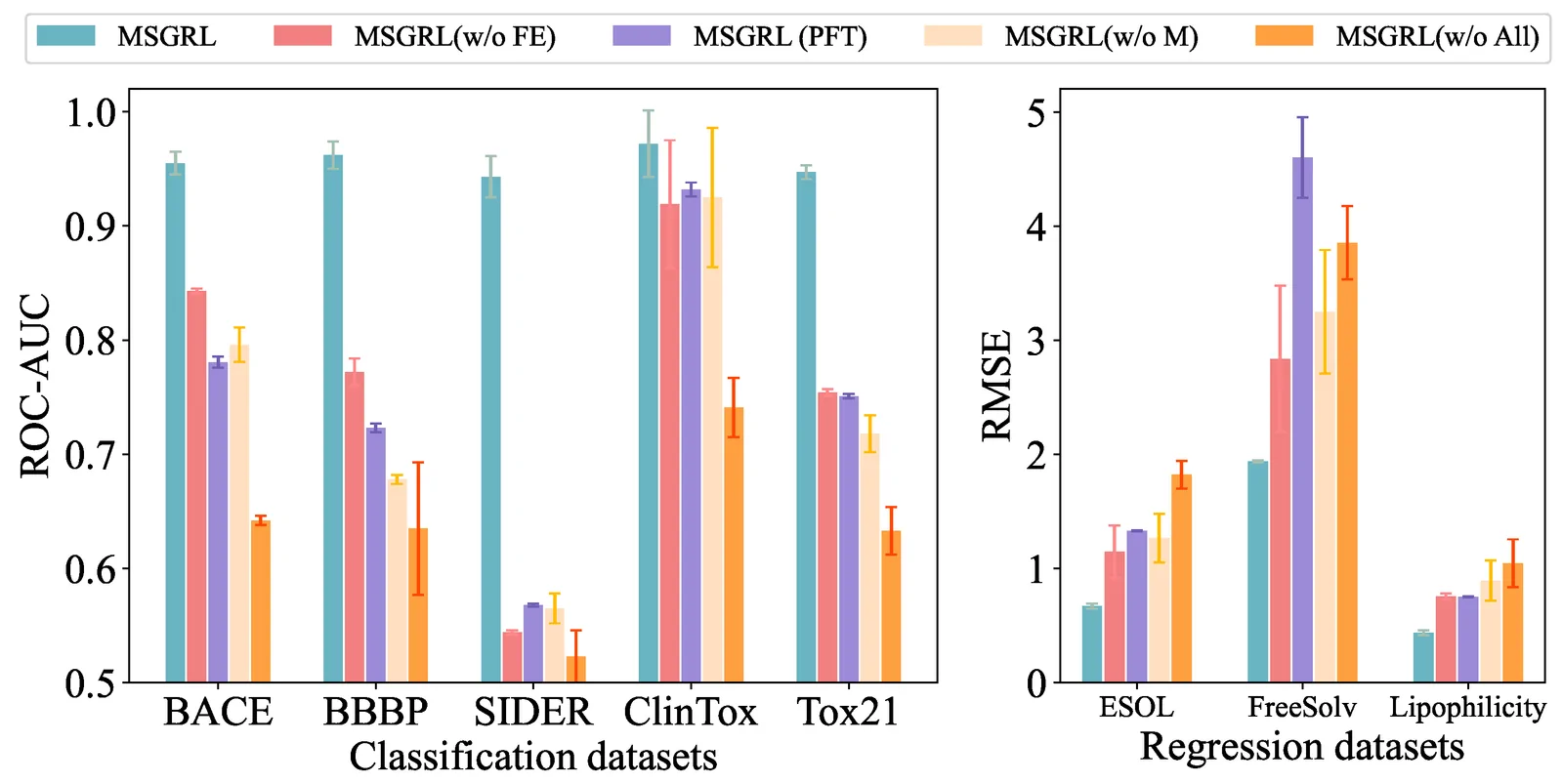
Systematic ablation analysis of MSGRL across eight MoleculeNet benchmark datasets under scaffold splitting with an 8:1:1 train/validation/test ratio. The full MSGRL model keeps the VMGAE-pretrained intra-motif encoder frozen during downstream training. PFT fine-tunes only the final message-passing block and its associated normalization layer, whereas w/o FE fine-tunes the entire pretrained encoder. The w/o M variant removes motif-based graph construction, and w/o All jointly removes the major architectural components. Bars and error bars represent the mean ± standard deviation over ten independent runs. ROC-AUC is reported for classification and RMSE for regression.

To evaluate the contribution of the major design choices in MSGRL, we conduct ablation experiments across all eight datasets. Figure 2 compares the full MSGRL model with four variants:

- **MSGRL with partial fine-tuning (PFT)** keeps the atom embedding and the first four message-passing blocks of the VMGAE-pretrained MPNN-GRU encoder frozen, while fine-tuning only the final message-passing block and its associated normalization layer.
- **MSGRL without frozen encoder (w/o FE)** fine-tunes the entire pretrained MPNN-GRU encoder together with the downstream prediction module.
- **MSGRL without motif-based graph construction (w/o M)** bypasses motif extraction and motif-based graph construction and performs prediction directly on the conventional atom-level molecular graph.
- **MSGRL without all key components (w/o All)** jointly removes the frozen-encoder strategy, motif-based graph construction, GRU-based message updating, and attention pooling, resulting in a simplified atom-level graph predictor.

**Effect of freezing the pretrained encoder.** To further examine the role of encoder freezing, we compare three downstream adaptation strategies: complete freezing in the full MSGRL model, partial fine-tuning (PFT), and full fine-tuning (w/o FE). In the PFT variant, the atom embedding and the first four message-passing blocks of the pretrained MPNN-GRU encoder remain frozen, while only the final message-passing block and its associated normalization layer are updated during downstream training. In the w/o FE variant, the entire pretrained encoder is fine-tuned together with the downstream prediction module.

As shown in Figure 2, complete freezing consistently provides the strongest performance among the three strategies. For example, the ROC-AUC on SIDER decreases from 0.943 with complete freezing to 0.568 with partial fine-tuning and 0.544 with full fine-tuning. On Tox21, the corresponding values are 0.947, 0.752, and 0.754. Similar degradation is also observed on BACE, BBBP, and the regression benchmarks. These results indicate that, under the evaluated settings, updating the pretrained intra-motif encoder does not improve downstream performance. A possible explanation is that downstream optimization shifts the representations learned from the self-supervised intra-motif reconstruction objective toward task-specific patterns. Accordingly, keeping the pretrained encoder fixed provides a stable structural basis for subsequent inter-motif learning.

**Effect of motif-based graph construction.** To assess the contribution of the hierarchical motif representation, we compare MSGRL with the w/o M variant, which bypasses motif extraction and performs prediction directly on the conventional atom-level molecular graph. As shown in Figure 2, removing motif-based graph construction consistently reduces performance. For example, the ROC-AUC decreases from 0.955 to 0.796 on BACE and from 0.972 to 0.925 on ClinTox. These results indicate that explicitly organizing molecular representations at the motif level provides complementary structural information beyond conventional atom-level connectivity.

**Overall contribution of the key components.** The w/o All variant jointly removes the frozen-encoder strategy, motif-based graph construction, GRU-based message updating, and attention pooling. This variant yields the weakest overall performance. Together with the individual ablations, these results show that the performance of MSGRL arises from the combined contributions of pretrained intra-motif representations and hierarchical motif-level downstream modeling rather than from a single component.

### 2.4. Effect of Graph-Level Pooling Modules

**Figure 3:**
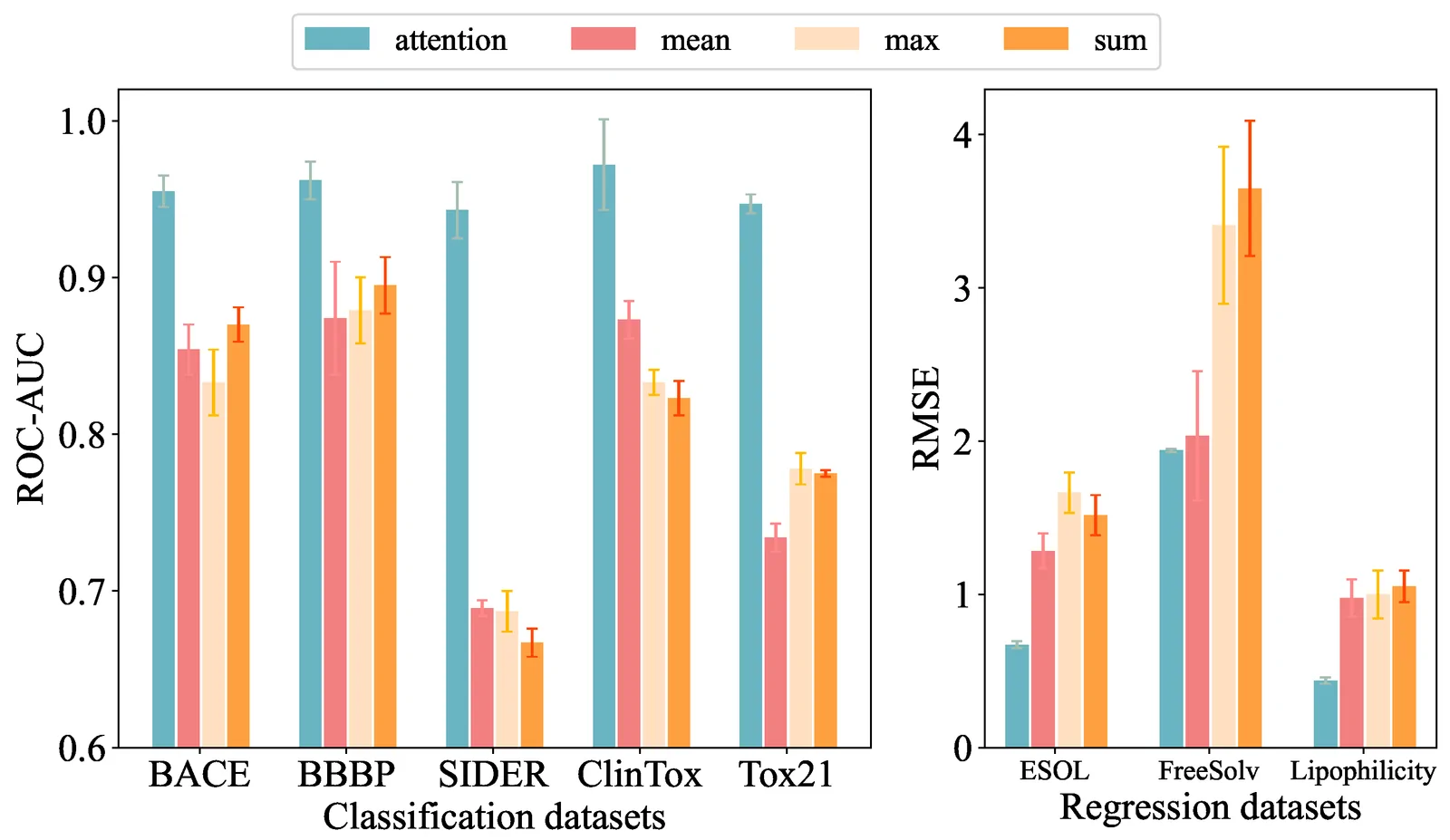
Comparison of graph-level pooling strategies in MSGRL across eight MoleculeNet datasets. Attention, mean, max, and sum pooling are used to aggregate motif-node representations into a molecular-level representation while all other model components are kept unchanged. Attention-based pooling provides the most stable overall performance across the evaluated tasks and is therefore used as the default readout strategy in MSGRL.

To examine how different graph-level readout strategies affect downstream prediction, we compare four pooling operations: attention, mean, max, and sum pooling. These pooling operations are applied to aggregate the motif-node representation matrix into a molecular-level representation $h_{g}$. The results are shown in Figure 3.

Overall, attention-based pooling achieves the most stable performance across the eight datasets. For classification tasks, it obtains the best ROC-AUC scores on BACE and BBBP, reaching 0.955 and 0.962, respectively. For regression tasks, attention-based pooling achieves RMSE values of 0.438 on Lipophilicity and 1.939 on FreeSolv. These results indicate that adaptively weighting motif nodes is beneficial for molecular property prediction, especially when different motifs contribute unequally to the target property.

The advantage of attention-based pooling lies in its ability to assign task-dependent weights to motif-node representations. Unlike mean, max, and sum pooling, which aggregate all motif nodes using fixed rules, attention pooling can emphasize motifs that are more relevant to the prediction task. This property is particularly important for molecular graphs, where only a subset of functional groups or substructures may dominate a specific biological or physicochemical property. For example, sum pooling may overemphasize molecules with more motif nodes, while max pooling may retain only the most dominant local signal and ignore complementary motif-level information.

Nevertheless, simpler pooling operations also provide competitive results on some regression datasets, indicating that the advantage of attention pooling is not uniform across all tasks. Overall, attention-based pooling provides a flexible readout mechanism and shows the most stable performance across the evaluated datasets; it is therefore adopted as the default graph-level pooling strategy in MSGRL.

### 2.5. Motif-Level Visualization Case Study

**Figure 4:**
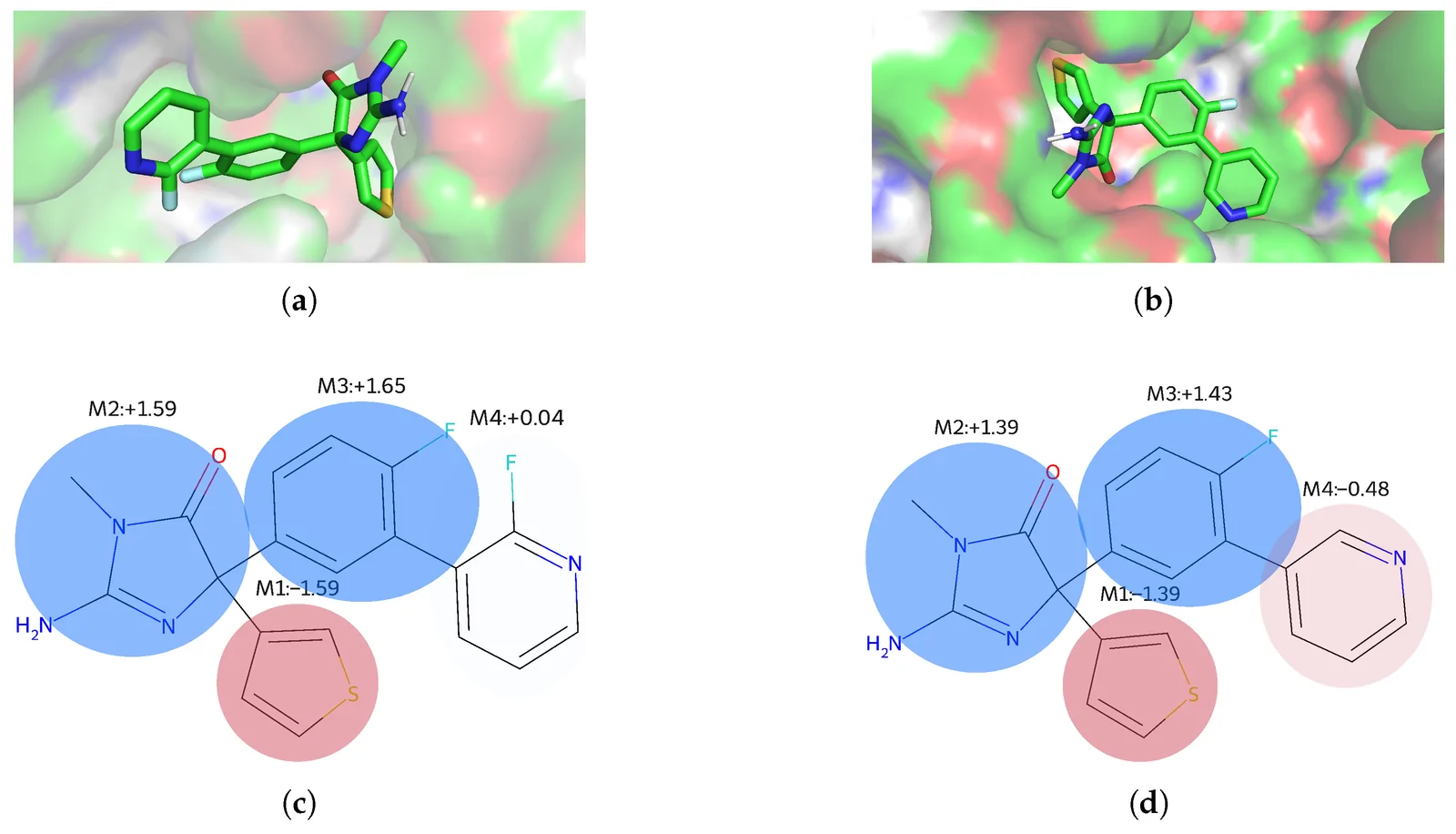
Comparison of docking poses and MSGRL-derived motif-level contribution maps for two representative molecules from the BACE dataset. (**a**,**b**) Predicted binding poses of BACE_598 and BACE_803 in the BACE1 binding pocket, respectively. (**c**,**d**) Corresponding two-dimensional motif-level contribution maps produced by MSGRL. Blue ellipses indicate positive contributions to the predicted active class, whereas orange ellipses indicate negative contributions; ellipse opacity is proportional to the absolute magnitude of the motif contribution score. For BACE_598, the docking score is −8.47, the prediction logit is 1.402, and p=0.802. For BACE_803, the corresponding values are −5.83, 0.667, and p=0.661. Here, the logit denotes the raw output of the prediction head before sigmoid transformation, and *p* denotes the predicted probability of the active class. (**a**) BACE_598 binding pose. (**b**) BACE_803 binding pose. (**c**) BACE_598 motif contribution. (**d**) BACE_803 motif contribution.

To further examine the chemical interpretability of MSGRL, we select two structurally related compounds from the BACE dataset, BACE_598 and BACE_803. Here, these names correspond to the compound identifiers used in the dataset; the underscore and following number are integral parts of each identifier rather than subscripts. As shown in Figure 4, each molecule is visualized from two complementary perspectives: its docked 3D binding pose in the BACE1 active site and its corresponding motif-level contribution map generated by MSGRL. This visualization strategy follows the drug–target interaction visualization scheme used in DeepDTAGen [28]. By jointly examining the binding conformation and motif-level attribution, we aim to assess whether the motif importance estimated by MSGRL is consistent with the local chemical environment of the ligand in the catalytic pocket.

The 3D binding poses are visualized using PyMOL (version 3.0.0) [29], and docking scores are obtained using AutoDock Vina (version 1.2.5) [30]. A more negative docking score indicates a more favorable predicted binding conformation. In parallel, MSGRL produces a signed motif-level contribution score $s_{i}=h_{\mathrm{gate},i}w^{⊤}{\mathrm{MLP}}_{\mathrm{pool}}\left(h_{m,i}^{\left(L_{m}\right)}\right)$ for each motif, where w denotes the weight vector of the prediction head. Motifs with $|s_{i}|\geq 0.01$ are selected for visualization. In the 2D molecular contribution maps, selected motifs are highlighted using semi-transparent ellipses: blue denotes a positive contribution to the predicted active class, whereas orange denotes a negative contribution. The opacity of each ellipse is proportional to $|s_{i}|$. The reported logit denotes the raw output of the prediction head before sigmoid transformation, and *P* denotes the sigmoid-transformed predicted probability of the molecule belonging to the positive class.

As shown in Figure 4a,c, the contribution map of BACE_598 identifies two central heteroaromatic motifs, M2 and M3, as the dominant positive contributors. Although the 3D binding pose itself does not determine the sign of the model attribution, it shows that these motifs are deeply positioned within the BACE1 binding pocket. This spatial placement is consistent with their large positive contribution scores and with the favorable docking score of −8.47, suggesting a comparatively favorable predicted binding configuration. In contrast, the sulfur-containing five-membered ring M1 shows a negative contribution. Its orientation toward a more solvent-exposed region in the binding pose is qualitatively consistent with its negative motif-level attribution.

For BACE_803, the contribution map shows that the two central motifs, M2 and M3, remain the major positive contributors, as shown in Figure 4b,d. Their spatial arrangement in the hydrophobic groove is similar to that observed for BACE_598, indicating that MSGRL assigns consistent importance to conserved central substructures across structurally related molecules. However, an important difference is observed at the terminal ring. Compared with BACE_598, the M4 motif of BACE_803 lacks the fluorine substituent and receives a more negative contribution score, changing from a weak positive contribution in BACE_598 to a negative contribution in BACE_803. In the corresponding 3D binding pose, this terminal ring is oriented toward the exterior of the binding pocket and appears to form fewer stabilizing contacts with the surrounding residues. Consistently, BACE_803 has a weaker docking score of −5.83, suggesting a less favorable predicted binding conformation than BACE_598.

Overall, this case study indicates that MSGRL can capture chemically meaningful motif-level differences between structurally related molecules. To complement the qualitative observations from these individual cases, we further evaluate motif-level attribution by analyzing the top-10 positive and negative contributing motifs across all benchmark datasets (Supplementary Figures S2–S17). Across these datasets, the resulting motif rankings highlight chemically plausible functional groups, such as polar groups in the solubility benchmarks and lipophilic aromatic rings in the Lipophilicity dataset. The motif-level contribution maps are not intended to replace structure-based docking analysis, but they provide complementary interpretability by linking model predictions to specific substructures. The observed consistency between the model-derived motif attributions and the docking-based binding poses provides complementary support for motif-level interpretation.

## 3. Discussion

MSGRL addresses the scarcity of motif-level supervision by learning motif representations through self-supervised reconstruction of motif-specific atom-based graphs. By combining motif-based graph construction, an MPNN-GRU encoder, VMGAE pretraining, and attention-based pooling, MSGRL provides a hierarchical representation framework that captures both intra-motif atomic structure and inter-motif organization. The experimental results show that MSGRL performs strongly on molecular classification tasks, achieving the highest ROC-AUC on all five classification datasets. The strong results on SIDER and Tox21 are consistent with the effectiveness of the frozen-encoder strategy observed in the ablation study. The ablation studies further show that updating the pretrained encoder during downstream training reduces performance under the evaluated settings, while removing motif-based graph construction also leads to performance degradation.

The motif-based graph construction strategy is central to the effectiveness of MSGRL. Compared with conventional atom-level graph modeling, it introduces chemically meaningful structural priors by organizing atoms into motifs and modeling their connectivity through shared atoms. This design is well aligned with the role of functional groups and recurring substructures in molecular property prediction. In addition, attention-based pooling enables the model to emphasize task-relevant motifs and provides motif-level importance cues for interpretation.

The visualization case study on the BACE dataset further suggests that the motif-level attributions produced by MSGRL are chemically meaningful. The contribution maps highlight conserved central motifs and capture substituent-level differences between structurally related molecules, showing consistency with the corresponding docking poses and docking scores. These results support the interpretability of MSGRL, although such visual evidence should be regarded as complementary to experimental validation. In particular, model-derived motif attribution scores reflect feature importance within the learned network rather than definitive physical binding mechanisms, and should be viewed as hypothesis-generating indicators alongside dataset-wide motif rankings. Several limitations remain. First, the current motif construction strategy is based on chemically motivated decomposition rules rather than experimentally annotated functional motifs. Second, MSGRL mainly uses 2D graph-based structural information and does not explicitly model 3D geometry or conformational flexibility. Third, although MSGRL performs strongly on classification tasks and achieves the lowest RMSE on Lipophilicity, continuous physicochemical property prediction may require additional descriptors or spatial information. These limitations point to possible extensions involving curated motif knowledge, 3D molecular geometry, and more specialized regression-oriented modeling.

## 4. Materials and Methods

### 4.1. Datasets

We select eight benchmark datasets for molecular property prediction from MoleculeNet [24] to evaluate the performance of MSGRL on both classification and regression tasks. These datasets cover diverse molecular properties related to biophysics, physical chemistry, physiology, toxicity, and side effects, enabling evaluation across different molecular property tasks. Specifically, five datasets are used for classification tasks, including BACE [31], BBBP [32], ClinTox [33,34], SIDER [35], and Tox21 [36]. The remaining three datasets are used for regression tasks, including ESOL [37], FreeSolv [38], and Lipophilicity, which is curated from the ChEMBL database [39]. Additional details on the datasets are provided in Supplementary Table S5. Together, these datasets enable a systematic evaluation of MSGRL on both discrete label prediction and continuous property prediction tasks.

### 4.2. Preliminaries

Given a molecular dataset $D={\left\{\left(M_{r},y_{r}\right)\right\}}_{r=1}^{N}$, where $M_{r}$ denotes the *r*-th molecule and $y_{r}$ denotes its corresponding property label, the goal of molecular property prediction is to learn a mapping function $f_{\theta }\left(\cdot \right)$ that predicts molecular properties from graph-based structural representations of molecules. For classification tasks, $y_{r}\in {\left\{0,1\right\}}^{T}$ denotes the binary labels over *T* prediction tasks, and the model outputs ${\hat{y}}_{r}\in {\left[0,1\right]}^{T}$. For regression tasks, $y_{r}\in R^{T}$ denotes continuous molecular properties, and the model outputs ${\hat{y}}_{r}\in R^{T}$.

In MSGRL, each molecule is first decomposed using BRICS into an initial set of molecular fragments, which are subsequently organized into a motif-based graph. In this study, we use the term “motif” operationally to denote a BRICS-derived substructure that serves as a node in the higher-level molecular representation. This terminology does not imply that every extracted substructure corresponds to a universally defined or experimentally annotated chemical motif. Rather, each such substructure retains its internal atom-level topology and is modeled together with its relationships to other substructures in the parent molecule. The motif-based graph is defined as $G_{m}=\left(V_{m},E_{m}\right)$, where $V_{m}={\left\{v_{p}^{m}\right\}}_{p=1}^{N_{m}}$ denotes the set of motif nodes and $N_{m}$ is the number of motifs in the molecule. Each motif node $v_{p}^{m}$ corresponds to a BRICS-derived substructure composed of one or more atoms. Let $A(v_{p}^{m})$ denote the set of atoms contained in motif $v_{p}^{m}$. When a BRICS-derived fragment *F* contains multiple sub-motifs, we first identify a shared-atom set $A_{\mathrm{shared}}\left(F\right)$. For each sub-motif $U_{p}$ derived from the same fragment, its atom set is constructed as

(1) $$\begin{array}{c} A\left(v_{p}^{m}\right)=U_{p}\cup A_{\mathrm{shared}}\left(F\right). \end{array}$$

**Figure 5:**
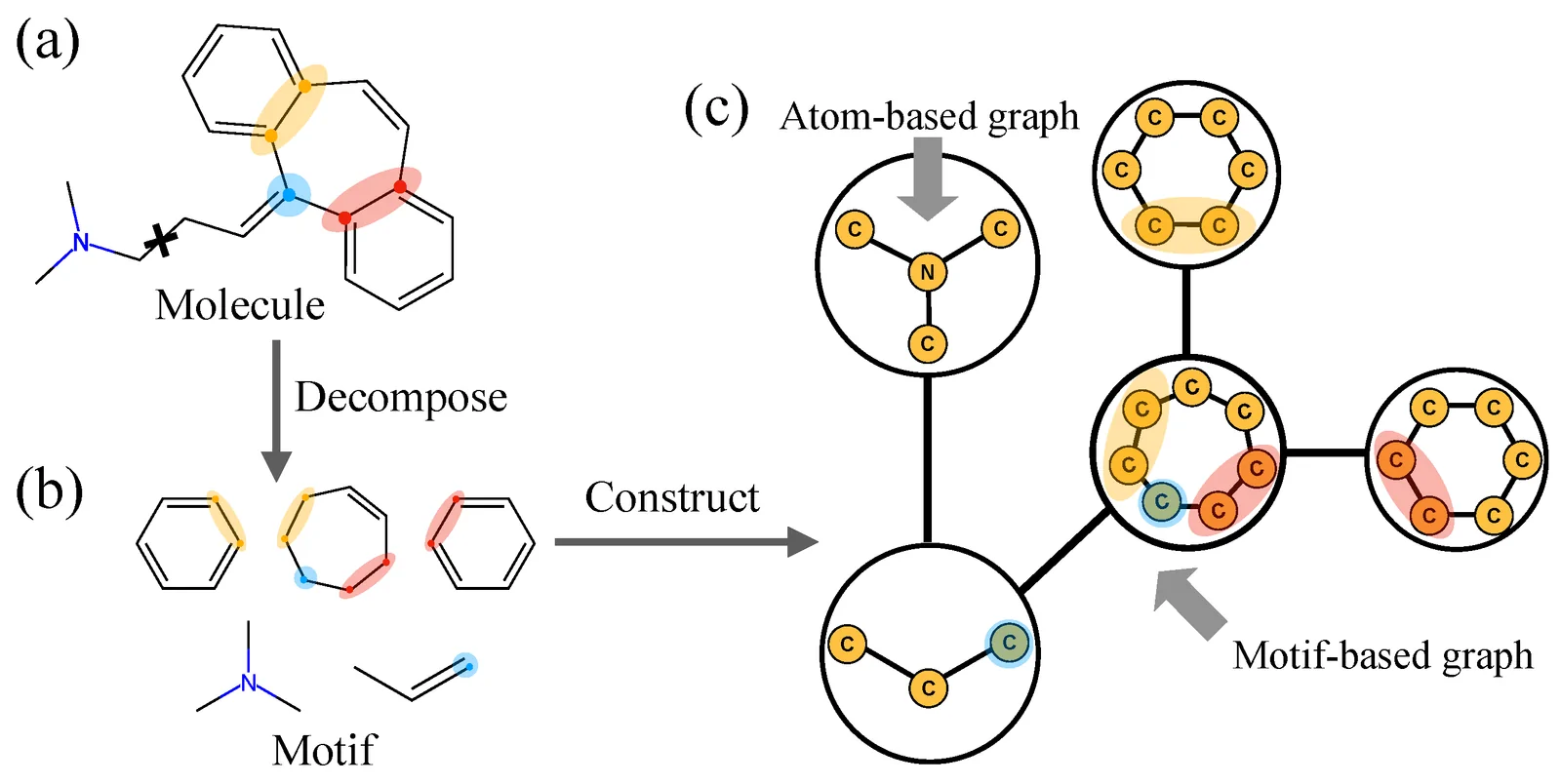
Illustration of BRICS-based motif extraction and hierarchical graph construction in MSGRL. (**a**) The input molecule is decomposed into BRICS-derived substructures that are treated as motif units. (**b**) Each motif retains its internal atom–bond topology and is represented as an atom-based graph; shared atoms are retained across the corresponding sub-motifs, resulting in overlap between motif-specific atom graphs. (**c**) A higher-level motif-based graph is constructed by treating individual motifs as nodes and defining inter-motif edges according to their shared atoms. Together, the two graph levels encode intra-motif atomic structure and inter-motif molecular organization.

Therefore, the shared atoms are retained in each sub-motif derived from *F*, and the resulting motif-specific atom graphs may overlap at these atoms. The edge set $E_{m}$ describes the connectivity between motif nodes based on shared atoms. For a pair of motif nodes $v_{p}^{m}$ and $v_{q}^{m}$, their shared atom set is defined as $S_{pq}=A\left(v_{p}^{m}\right)\cap A\left(v_{q}^{m}\right)$. If $S_{pq}\neq \emptyset$, the corresponding inter-motif edge set is denoted as $E_{pq}^{m}={\left\{e_{pq,k}^{m}\right\}}_{k=1}^{K_{pq}}$, where each $e_{pq,k}^{m}$ is associated with a shared atom between the two motifs, and $K_{pq}=\left|S_{pq}\right|$ denotes the number of shared atoms between $v_{p}^{m}$ and $v_{q}^{m}$. Because the same shared atom is retained in multiple motif-specific graphs, this construction introduces limited representational redundancy. These occurrences refer to the same atom in the parent molecule and retain the same initial atom attributes. However, they are encoded within different motif contexts and may therefore acquire context-dependent representations. This overlap is intentionally introduced to retain shared structural context between related motifs. Nevertheless, this construction may increase the representational exposure of shared atoms. In addition, BRICS decomposition imposes a structural prior. We therefore acknowledge both effects as potential sources of methodological bias. Since the construction depends only on molecular structure and not on downstream labels, it does not introduce label leakage. The process of constructing motif-based graphs from molecules is illustrated in Figure 5, and the detailed construction procedure is summarized in Algorithm 1.

Each motif is further represented by an atom-based graph that characterizes its internal atomic structure. Specifically, the atom-based graph associated with motif $v_{p}^{m}$ is denoted as $G_{a,p}=\left(V_{a,p},E_{a,p}\right)$, where $V_{a,p}={\left\{v_{p,i}^{a}\right\}}_{i=1}^{N_{a,p}}$ denotes the set of atom nodes in the *p*-th motif and $N_{a,p}$ is the number of atoms in this motif. Each atom node $v_{p,i}^{a}$ represents an individual atom, and $E_{a,p}=\left\{e_{p,ij}^{a}|v_{p,i}^{a},v_{p,j}^{a}\in V_{a,p}\right\}$ denotes the set of chemical bonds within the motif. For a molecule with $N_{m}$ motifs, we denote the collection of its atom-based graphs as $G_{a}={\left\{G_{a,p}\right\}}_{p=1}^{N_{m}}$. For notational simplicity, $G_{a}=\left(V_{a},E_{a}\right)$ is used to denote a generic atom-based graph when the motif index is clear from the context.
**Algorithm 1** Motif-based graph construction**Require:** Molecule M**Ensure:** Motif-based graph $G_{m}=\left(V_{m},E_{m}\right)$ and atom-based graph collection $G_{a}={\left\{G_{a,p}\right\}}_{p=1}^{N_{m}}$  1:$V_{m}⇐\emptyset$, $E_{m}⇐\emptyset$, $G_{a}⇐\emptyset$  2:p⇐0  3:F⇐BRICS(M)    ▹ Generate initial fragments using the BRICS algorithm [40]  4:**for** each fragment F∈F **do**  5:    **if** *F* contains sub-motifs **then**  6:        $A_{\mathrm{shared}}⇐\mathrm{ExtractSharedAtoms}\left(F\right)$  7:        $U⇐\mathrm{ExtractSubMotifs}(F\setminus A_{\mathrm{shared}})$  8:        **for** each sub-motif U∈U **do**  9:           p⇐p+110:           $A\left(v_{p}^{m}\right)⇐U\cup A_{\mathrm{shared}}$     ▹ Retain the shared-atom set in each sub-motif derived from the same fragment11:           $v_{p}^{m}⇐\mathrm{CreateMotifNode}\left(A\left(v_{p}^{m}\right)\right)$12:           $G_{a,p}⇐\mathrm{InducedAtomGraph}\left(M,A\left(v_{p}^{m}\right)\right)$13:           $V_{m}⇐V_{m}\cup \left\{v_{p}^{m}\right\}$14:           $G_{a}⇐G_{a}\cup \left\{G_{a,p}\right\}$15:        **end for**16:    **else**17:        p⇐p+118:        $A(v_{p}^{m})⇐F$19:        $v_{p}^{m}⇐\mathrm{CreateMotifNode}\left(A\left(v_{p}^{m}\right)\right)$20:        $G_{a,p}⇐\mathrm{InducedAtomGraph}\left(M,A\left(v_{p}^{m}\right)\right)$21:        $V_{m}⇐V_{m}\cup \left\{v_{p}^{m}\right\}$22:        $G_{a}⇐G_{a}\cup \left\{G_{a,p}\right\}$23:    **end if**24:**end for**25:**for** each pair of motif nodes $v_{p}^{m},v_{q}^{m}\in V_{m}$, p<q **do**26:    $S_{pq}⇐A\left(v_{p}^{m}\right)\cap A\left(v_{q}^{m}\right)$27:    **if** $S_{pq}\neq \emptyset$ **then**28:        $K_{pq}⇐\left|S_{pq}\right|$29:        $E_{pq}^{m}⇐\left\{e_{pq,k}^{m}|a_{k}\in S_{pq},\;k=1,\ldots ,K_{pq}\right\}$30:        $E_{m}⇐E_{m}\cup E_{pq}^{m}$31:    **end if**32:**end for**33:$G_{m}⇐\left(V_{m},E_{m}\right)$    **return** $G_{m},G_{a}$

### 4.3. The MPNN-GRU Encoder

The MPNN-GRU encoder operates on the atom-based graph of each motif to learn atom-level representations. Since the atom-based graph $G_{a,p}$ of each motif has been defined in Section 4.2, we formulate the encoder on a generic atom-based graph $G_{a}=\left(V_{a},E_{a}\right)$ for simplicity. Let $x_{a,i}\in R^{d_{n}}$ and $x_{a,ij}\in R^{d_{e}}$ denote the initial feature vectors of atom node $v_{i}^{a}$ and bond $e_{ij}^{a}$, respectively. The detailed descriptions of these atom and bond features are provided in Supplementary Tables S3 and S4. The initial hidden state of atom $v_{i}^{a}$ is obtained by mapping its input feature into the hidden space

(2) $$\begin{array}{c} h_{a,i}^{\left(0\right)}=\phi_{1}\left(W_{\mathrm{in}}x_{a,i}+b_{\mathrm{in}}\right), \end{array}$$

where $W_{\mathrm{in}}$ and $b_{\mathrm{in}}$ are trainable parameters, and $\phi_{1}\left(\cdot \right)$ denotes a nonlinear activation function.

At the *l*-th layer, the encoder first computes messages from neighboring atoms. For atom $v_{i}^{a}$, the message aggregated from its neighbors is defined as

(3) $$\begin{array}{c} m_{a,i}^{\left(l\right)}=\sum_{j\in N_{a}\left(i\right)}M_{a}\left(h_{a,i}^{\left(l\right)},h_{a,j}^{\left(l\right)},x_{a,ij}\right), \end{array}$$

where $N_{a}\left(i\right)$ denotes the set of neighboring atoms of $v_{i}^{a}$, and $M_{a}\left(\cdot \right)$ is an atom-level message function that integrates the representation of the central atom, the representation of a neighboring atom, and the corresponding bond feature. In our implementation, $M_{a}\left(\cdot \right)$ is parameterized by a learnable neural transformation

(4) $$\begin{array}{c} M_{a}\left(h_{a,i}^{\left(l\right)},h_{a,j}^{\left(l\right)},x_{a,ij}\right)=\phi_{2}\left(W_{m}^{\left(l\right)}\left[h_{a,i}^{\left(l\right)}∥h_{a,j}^{\left(l\right)}∥x_{a,ij}\right]+b_{m}^{\left(l\right)}\right), \end{array}$$

where ∥ denotes vector concatenation, $W_{m}^{\left(l\right)}$ and $b_{m}^{\left(l\right)}$ are trainable parameters, and $\phi_{2}\left(\cdot \right)$ is a nonlinear activation function.

The aggregated message is then used to update the atom representation through a GRU

(5) $$\begin{array}{c} h_{a,i}^{\left(l+1\right)}=\mathrm{GRU}\left(m_{a,i}^{\left(l\right)},h_{a,i}^{\left(l\right)}\right). \end{array}$$

The GRU-based update allows the encoder to recurrently refine atom representations across multiple message-passing layers while retaining useful information from previous hidden states.

After $L_{a}$ MPNN-GRU layers, the encoder outputs an atom-level representation matrix

(6) $$\begin{array}{c} H_{a}={\left[h_{a,1}^{\left(L_{a}\right)},h_{a,2}^{\left(L_{a}\right)},\ldots ,h_{a,N_{a}}^{\left(L_{a}\right)}\right]}^{⊤}\in R^{N_{a}\times d_{h}}. \end{array}$$

For the *p*-th motif, the corresponding output is denoted as $H_{a,p}\in R^{N_{a,p}\times d_{h}}$, which serves as the motif-level feature matrix composed of atom representations within motif $v_{p}^{m}$. The encoder producing this matrix is optimized by the VMGAE module during self-supervised pretraining and then kept frozen for downstream prediction.

### 4.4. The VMGAE Self-Supervised Pretraining Module

The VMGAE self-supervised pretraining module is designed to pretrain the MPNN-GRU encoder on motif-specific atom-based graphs without requiring explicit motif-level labels. Since supervised annotations for motif representation learning are generally unavailable, VMGAE learns atom-level latent representations by reconstructing the internal graph structure of each motif. This self-supervised objective encourages the encoder to capture chemical-bond connectivity within motifs, thereby providing structurally informative motif-level feature matrices for the downstream prediction module.

For the atom-based graph $G_{a,p}$ associated with motif $v_{p}^{m}$, the MPNN-GRU encoder generates an atom-level feature matrix $H_{a,p}\in R^{N_{a,p}\times d_{h}}$. For notational simplicity, we omit the motif index *p* and denote the feature matrix and adjacency matrix of a generic atom-based graph as $H_{a}$ and $A_{a}$, respectively. Following the variational graph autoencoder framework [25], VMGAE approximates the posterior distribution of latent atom representations as

(7) $$\begin{array}{c} q\left(Z_{a}|H_{a},A_{a}\right)=\prod_{i=1}^{N_{a}}q\left(z_{a,i}|H_{a},A_{a}\right), \end{array}$$

where $Z_{a}={\left[z_{a,1},z_{a,2},\ldots ,z_{a,N_{a}}\right]}^{⊤}$ denotes the latent representation matrix of atom nodes.

The posterior distribution is parameterized by two graph-aware projection heads, which estimate the mean and log-variance matrices from $H_{a}$ and $A_{a}$

(8) $$\begin{array}{c} \mu_{a}=f_{\mu }\left(H_{a},A_{a}\right), \end{array}$$

(9) $$\begin{array}{c} \log\sigma_{a}^{2}=f_{\sigma }\left(H_{a},A_{a}\right), \end{array}$$

where $f_{\mu }\left(\cdot \right)$ and $f_{\sigma }\left(\cdot \right)$ are learnable graph-aware transformations. The latent atom representation matrix is then sampled using the reparameterization trick

(10) $$\begin{array}{c} Z_{a}=\mu_{a}+\epsilon ⊙\exp\left(\frac{1}{2}\log\sigma_{a}^{2}\right),\;\epsilon ∼N\left(0,I\right), \end{array}$$

where ⊙ denotes element-wise multiplication.

To reconstruct the internal chemical bond topology of the atom-based graph, VMGAE adopts an inner-product decoder

(11) $$\begin{array}{c} {\hat{A}}_{a}=\sigma \left(Z_{a}Z_{a}^{⊤}\right), \end{array}$$

where σ(·) is the sigmoid activation function and ${\hat{A}}_{a}$ denotes the reconstructed adjacency matrix.

The VMGAE objective consists of two terms: an adjacency reconstruction loss and a KL divergence regularization term. The reconstruction term encourages $Z_{a}$ to preserve the local bond connectivity within each motif, while the KL term regularizes the posterior distribution toward a standard Gaussian prior

(12) $$\begin{array}{c} L_{\mathrm{VMGAE}}=L_{\mathrm{rec}}+L_{\mathrm{KL}}, \end{array}$$

where

(13) $$\begin{array}{c} L_{\mathrm{rec}}=-E_{q(Z_{a}|H_{a},A_{a})}\left[\log p(A_{a}|Z_{a})\right], \end{array}$$

and

(14) $$\begin{array}{c} L_{\mathrm{KL}}=\mathrm{KL}\left[q\left(Z_{a}|H_{a},A_{a}\right)∥p\left(Z_{a}\right)\right], \end{array}$$

with $p\left(Z_{a}\right)=N\left(0,I\right)$ denoting the standard Gaussian prior. Equivalently, this objective corresponds to minimizing the negative evidence lower bound. After VMGAE pretraining, the MPNN-GRU encoder is kept frozen and used to produce structurally informed atom-level feature matrices, which are subsequently pooled into motif-level representations for downstream molecular property prediction.

### 4.5. The Downstream Prediction Module

The downstream prediction module transforms VMGAE-pretrained motif-level feature matrices into molecular-level representations for property prediction. In this stage, the pretrained MPNN-GRU encoder is kept frozen, and only the motif-level message-passing network, attention pooling module, and task-specific prediction head are trained using downstream labels. Unlike the MPNN-GRU encoder and VMGAE module, which operate on motif-specific atom-based graphs, the downstream module performs message passing on the motif-based graph $G_{m}=\left(V_{m},E_{m}\right)$. For each motif node $v_{p}^{m}$, we use the frozen pretrained MPNN-GRU encoder to obtain a motif-level feature matrix $H_{a,p}\in R^{N_{a,p}\times d_{h}}$, where each row corresponds to the representation of an atom within the motif. This matrix is first pooled into a motif vector

(15) $$\begin{array}{c} h_{m,p}^{\left(0\right)}={\mathrm{Pool}}_{a}\left(H_{a,p}\right), \end{array}$$

where ${\mathrm{Pool}}_{a}\left(\cdot \right)$ denotes an atom-level pooling function over the atoms within motif $v_{p}^{m}$. The resulting vector $h_{m,p}^{\left(0\right)}\in R^{d_{h}}$ is used as the initial representation of motif node $v_{p}^{m}$.

For two connected motif nodes $v_{p}^{m}$ and $v_{q}^{m}$, their inter-motif edge set is denoted as $E_{pq}^{m}={\left\{e_{pq,k}^{m}\right\}}_{k=1}^{K_{pq}}$. The corresponding edge representation is obtained by aggregating the features associated with these inter-motif edges

(16) $$\begin{array}{c} {\bar{x}}_{m,pq}={\mathrm{Pool}}_{e}\left({\left\{x_{m,pq,k}\right\}}_{k=1}^{K_{pq}}\right), \end{array}$$

where ${\mathrm{Pool}}_{e}\left(\cdot \right)$ denotes an edge-level pooling function and $x_{m,pq,k}$ is the feature vector associated with edge $e_{pq,k}^{m}$.

The motif vectors are then updated through motif-level message passing. At the *l*-th layer, the message received by motif node $v_{p}^{m}$ is computed as

(17) $$\begin{array}{c} m_{m,p}^{\left(l\right)}=\sum_{q\in N_{m}\left(p\right)}M_{m}\left(h_{m,p}^{\left(l\right)},h_{m,q}^{\left(l\right)},{\bar{x}}_{m,pq}\right), \end{array}$$

where $N_{m}\left(p\right)$ denotes the set of neighboring motif nodes of $v_{p}^{m}$, and $M_{m}\left(\cdot \right)$ is the motif-level message function. The motif-node representation is then updated by a GRU-based recurrent update

(18) $$\begin{array}{c} h_{m,p}^{\left(l+1\right)}=\mathrm{GRU}\left(m_{m,p}^{\left(l\right)},h_{m,p}^{\left(l\right)}\right). \end{array}$$

Here, $m_{m,p}^{\left(l\right)}$ serves as the input to the GRU at the *l*-th message-passing layer, while $h_{m,p}^{\left(l\right)}$ is the previous hidden state of motif node $v_{p}^{m}$.

**Figure 6:**
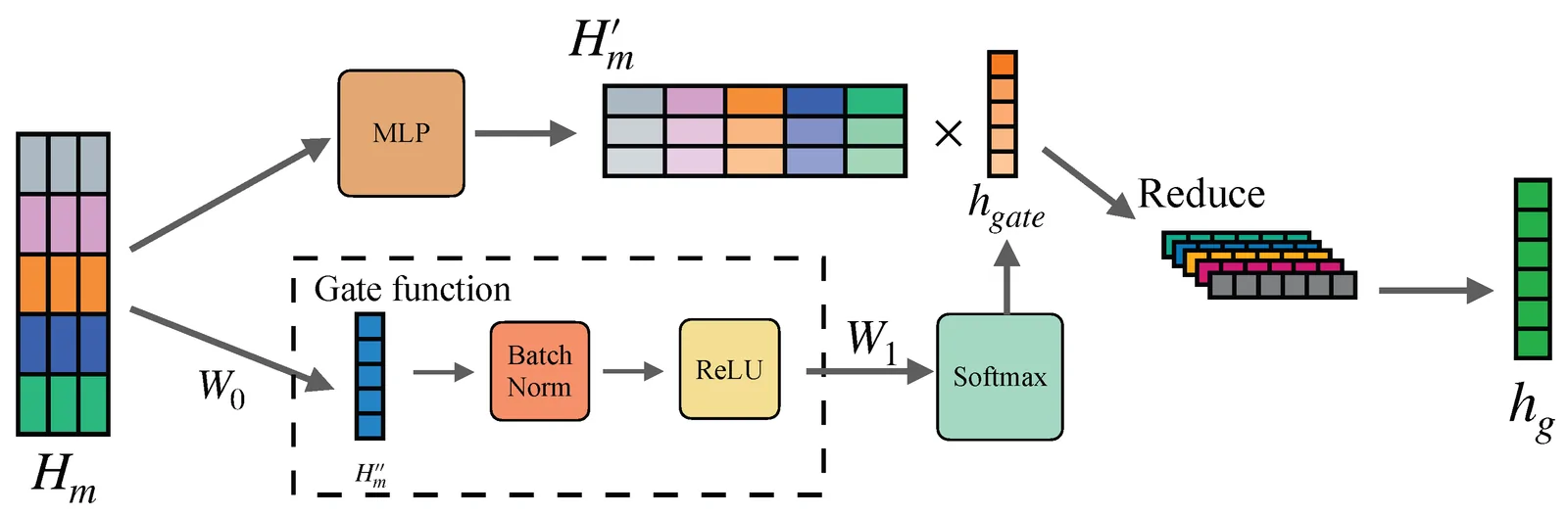
Illustration of the attention-based graph-level pooling mechanism in MSGRL. The motif-node representation matrix $H_{m}$ is transformed to obtain motif features and passed through a gating function followed by softmax normalization to generate adaptive attention weights. The transformed motif features are weighted according to these scores and aggregated into the molecular-level representation $h_{g}$, allowing motifs to contribute unequally to the final molecular representation.

After $L_{m}$ layers of motif-level message passing, an attention-based pooling mechanism is applied to obtain a molecular-level representation. As illustrated in Figure 6, the attention pooling module transforms the motif-node representation matrix into a molecular-level vector by assigning adaptive importance weights to different motif nodes. Specifically, it first computes a gating score for each motif representation and then uses the normalized attention weights to aggregate the transformed motif features into the final molecular representation $h_{g}$.

For motif node $v_{p}^{m}$, the attention score is computed as

(19) $$\begin{array}{c} g_{p}=w_{2}^{⊤}\phi_{3}\left(\mathrm{BatchNorm}\left(W_{1}h_{m,p}^{\left(L_{m}\right)}\right)\right), \end{array}$$

where $W_{1}$ and $w_{2}$ are trainable parameters, and $\phi_{3}\left(\cdot \right)$ denotes a nonlinear activation function. The normalized attention weight is then calculated by

(20) $$\begin{array}{c} h_{gate,p}=\frac{\exp(g_{p})}{\sum_{r=1}^{N_{m}}\exp\left(g_{r}\right)}. \end{array}$$

The molecular-level representation is obtained by aggregating motif-node representations according to their attention weights

(21) $$\begin{array}{c} h_{g}=\sum_{p=1}^{N_{m}}h_{gate,p}\;{\mathrm{MLP}}_{\mathrm{pool}}\left(h_{m,p}^{\left(L_{m}\right)}\right). \end{array}$$

The attention weights ${\left\{h_{gate,p}\right\}}_{p=1}^{N_{m}}$ provide motif-level importance cues, reflecting the relative contribution of different motifs to the final molecular representation.

Finally, the molecular-level representation $h_{g}$ is fed into a task-specific prediction head

(22) $$\begin{array}{c} o={\mathrm{MLP}}_{\mathrm{pred}}\left(h_{g}\right), \end{array}$$

where o denotes the raw output of the prediction head.

For classification tasks, the predicted probability is obtained by $\hat{y}=\sigma \left(o\right)$, and the model is optimized using the binary cross-entropy loss

(23) $$\begin{array}{c} L_{\mathrm{cls}}=-\frac{1}{\left|\Omega \right|}\sum_{(r,t)\in \Omega }\left[y_{r,t}\log{\hat{y}}_{r,t}+\left(1-y_{r,t}\right)\log\left(1-{\hat{y}}_{r,t}\right)\right], \end{array}$$

where Ω denotes the set of molecule–task pairs with available labels, and $y_{r,t}$ and ${\hat{y}}_{r,t}$ denote the ground-truth label and predicted probability for the *t*-th task of the *r*-th molecule, respectively.

For regression tasks, the raw output is directly used as the prediction, i.e., $\hat{y}=o$. The model is optimized using the mean squared error loss

(24) $$\begin{array}{c} L_{\mathrm{reg}}=\frac{1}{\left|\Omega \right|}\sum_{(r,t)\in \Omega }{\left({\hat{y}}_{r,t}-y_{r,t}\right)}^{2}, \end{array}$$

where $y_{r,t}$ and ${\hat{y}}_{r,t}$ denote the ground-truth value and predicted value for the *t*-th regression task of the *r*-th molecule, respectively. The final supervised objective is selected according to the task type of each dataset.

### 4.6. Experimental Settings

Each dataset is partitioned into training, validation, and test sets using scaffold-based splitting with an 8:1:1 ratio [18,20]. Molecules assigned to the same scaffold group are kept within the same partition to reduce scaffold overlap between training and evaluation data. Molecular structures are processed using RDKit, and the atom and bond features used by MSGRL follow the Open Graph Benchmark (OGB) feature definitions. ROC-AUC is used as the evaluation metric for classification datasets, whereas RMSE is used for regression datasets.

For each downstream dataset, VMGAE pretraining is performed exclusively on molecules in the corresponding training split. Neither validation nor test molecules are used for self-supervised pretraining or supervised parameter optimization. The validation set is used for model selection and, where applicable, hyperparameter selection, whereas the test set is used only for final evaluation.

Both the intra-motif MPNN-GRU encoder and the downstream motif-graph propagation network contain five message-passing layers, with a hidden dimension of 300 and a dropout rate of 0.5. VMGAE pretraining is performed for 400 epochs using Adam with a learning rate of $1\times 10^{-4}$ and a batch size of 32. During downstream training, the pretrained MPNN-GRU encoder is frozen (requires_grad=False), while the motif-graph network, attention-pooling module, and prediction head are optimized for 400 epochs using the same learning rate and batch size.

For baseline comparison, all methods use the same dataset versions, scaffold-based partitions, evaluation metrics, and test-set isolation protocol. Architecture-specific preprocessing, input representations, and hyperparameters follow the corresponding official implementations or original publications whenever available; when multiple recommended configurations are available, selection is based only on validation performance. All methods are evaluated over ten runs with different random seeds, and results are reported as mean ± standard deviation. All experiments are implemented in PyTorch (version 2.7.1) [41] and executed on an Ubuntu server equipped with an NVIDIA GeForce RTX 5090 GPU. Statistical comparisons between MSGRL and the strongest competing baseline on each dataset are performed using two-sided Welch’s *t*-tests based on the ten-run summary statistics, with Holm correction applied for multiple comparisons. Adjusted *p*-values below 0.05 are considered statistically significant.

## 5. Conclusions

In this study, we propose MSGRL, a motif-driven self-supervised graph representation learning framework for molecular property prediction. MSGRL decomposes molecules into molecular fragments, represents each motif as an atom-based graph, and constructs a motif-based graph to model inter-motif connectivity. The MPNN-GRU encoder is pretrained through a VMGAE objective and then kept frozen to provide stable motif-level feature matrices for downstream prediction.

The downstream prediction module further pools motif-level feature matrices into motif vectors, performs message passing on the motif-based graph, and applies attention-based pooling to obtain molecular-level representations. This design enables MSGRL to preserve the structural features learned during label-independent pretraining while learning task-specific predictors from downstream labels.

Experiments on eight MoleculeNet benchmark datasets demonstrate the effectiveness of MSGRL. The model achieves the highest ROC-AUC scores on all five classification datasets and the lowest RMSE on Lipophilicity, while its performance on ESOL and FreeSolv is more mixed. Ablation studies confirm the importance of the frozen pretrained encoder, motif-based graph construction, and attention-based pooling. Motif-level attribution analyses provide qualitative and dataset-level evidence regarding the substructures emphasized by the model and offer complementary support for model interpretation.

Future work will extend MSGRL by incorporating curated motif annotations, explicit 3D molecular geometry, and more complex biochemical reasoning scenarios, such as drug–drug interaction prediction, drug–target affinity estimation, and retrosynthesis analysis. These directions may further improve the generality, interpretability, and practical utility of motif-driven self-supervised molecular representation learning.

## Figures and Tables

**Table 1:** Performance comparison of MSGRL and baseline methods on eight MoleculeNet datasets using scaffold-based train/validation/test splitting. Results are reported as mean ± standard deviation over ten runs with different random seeds.

Task	Classification (ROC-AUC, ↑)					Regression (RMSE, ↓)		
**Dataset**	**BACE**	**BBBP**	**SIDER**	**ClinTox**	**Tox21**	**ESOL**	**FreeSolv**	**Lipophilicity**
GCN-MPNN	0.756 ± 0.014	0.651 ± 0.018	0.583 ± 0.003	0.941 ± 0.006	0.748 ± 0.074	1.224 ± 0.036	2.856 ± 0.162	0.747 ± 0.032
GIN-MPNN	0.793 ± 0.047	0.663 ± 0.012	0.579 ± 0.015	0.854 ± 0.054	0.733 ± 0.015	1.414 ± 0.101	4.230 ± 0.371	0.737 ± 0.021
AttentiveFP	0.758 ± 0.015	0.663 ± 0.019	0.581 ± 0.017	0.597 ± 0.053	0.712 ± 0.008	1.280 ± 0.068	2.940 ± 0.143	1.021 ± 0.007
GPS	0.766 ± 0.035	0.660 ± 0.010	0.626 ± 0.009	0.820 ± 0.040	0.744 ± 0.009	1.036 ± 0.248	2.729 ± 0.923	0.794 ± 0.023
DeeperGCN	0.810 ± 0.030	0.690 ± 0.016	0.560 ± 0.006	0.925 ± 0.002	0.750 ± 0.001	1.097 ± 0.121	2.296 ± 0.261	0.762 ± 0.171
VGAE	0.610 ± 0.045	0.612 ± 0.008	0.539 ± 0.013	0.502 ± 0.064	0.663 ± 0.007	1.444 ± 0.025	3.969 ± 0.286	1.096 ± 0.009
MICRO-Graph	0.772 ± 0.020	0.843 ± 0.010	0.566 ± 0.008	0.780 ± 0.009	0.770 ± 0.007	0.924 ± 0.077	2.140 ± 0.162	0.798 ± 0.029
MGSSL	0.805 ± 0.072	0.845 ± 0.011	0.591 ± 0.016	0.877 ± 0.021	0.788 ± 0.022	1.935 ± 0.332	2.890 ± 0.837	0.951 ± 0.192
HiMol	0.840 ± 0.072	0.865 ± 0.064	0.593 ± 0.022	0.702 ± 0.092	0.815 ± 0.010	0.986 ± 0.231	2.731 ± 0.440	0.716 ± 0.254
GemNet	0.746 ± 0.013	0.617 ± 0.011	0.606 ± 0.018	0.823 ± 0.062	0.751 ± 0.023	0.913 ± 0.028	1.985 ± 0.113	0.747 ± 0.018
MetaGIN	0.690 ± 0.042	0.917 ± 0.018	0.645 ± 0.024	0.908 ± 0.081	0.830 ± 0.001	0.780 ± 0.061	1.397 ± 0.062	0.532 ± 0.013
HimGNN	0.812 ± 0.088	0.901 ± 0.054	0.622 ± 0.042	0.902 ± 0.044	0.811 ± 0.023	0.890 ± 0.231	1.901 ± 0.514	0.714 ± 0.203
SCI	0.830 ± 0.116	0.730 ± 0.029	0.715 ± 0.005	0.922 ± 0.025	0.802 ± 0.007	0.639 ± 0.004	1.948 ± 2.015	0.508 ± 0.011
FraGAT	0.927 ± 0.002	0.933 ± 0.006	0.673 ± 0.011	0.969 ± 0.001	0.863 ± 0.005	**0.478 ± 0.012**	**0.538 ± 0.022**	0.569 ± 0.031
MSGRL	**0.955 ± 0.010**	**0.962 ± 0.012**	**0.943 ± 0.018**	**0.972 ± 0.029**	**0.947 ± 0.006**	0.671 ± 0.003	1.939 ± 0.010	**0.438 ± 0.021**

Note: ↑ means that the higher result is better and ↓ means that the lower result is better. The highest mean value in each column is shown in bold, and the second-highest mean value is underlined for classification tasks. For regression tasks, the lowest and second-lowest mean values are shown in bold and underlined, respectively.

## Data Availability

No new datasets were generated during the current study. All benchmark datasets used in this study are publicly available from MoleculeNet and their original sources, as cited in the manuscript. The source code for MSGRL is available at https://github.com/WUYOU079/MSGRL (accessed on 25 August 2026).

## Supplementary Materials

The following supporting information can be downloaded at https://www.mdpi.com/article/10.3390/molecules31173008/s1, Figure S1: Prediction–ground-truth agreement and distributional comparison of MSGRL on the three regression benchmarks; Figure S2: Top 10 molecular motifs with the highest positive contribution scores on the BACE dataset; Figure S3: Top 10 molecular motifs with the most negative contribution scores on the BACE dataset; Figure S4: Top 10 molecular motifs with the highest positive contribution scores on the BBBP dataset; Figure S5: Top 10 molecular motifs with the most negative contribution scores on the BBBP dataset; Figure S6: Top 10 molecular motifs with the highest positive contribution scores on the SIDER dataset; Figure S7: Top 10 molecular motifs with the most negative contribution scores on the SIDER dataset; Figure S8: Top 10 molecular motifs with the highest positive contribution scores on the ClinTox dataset; Figure S9: Top 10 molecular motifs with the most negative contribution scores on the ClinTox dataset; Figure S10: Top 10 molecular motifs with the highest positive contribution scores on the Tox21 dataset; Figure S11: Top 10 molecular motifs with the most negative contribution scores on the Tox21 dataset; Figure S12: Top 10 molecular motifs with the highest positive contribution scores on the ESOL dataset; Figure S13: Top 10 molecular motifs with the most negative contribution scores on the ESOL dataset; Figure S14: Top 10 molecular motifs with the highest positive contribution scores on the FreeSolv dataset; Figure S15: Top 10 molecular motifs with the most negative contribution scores on the FreeSolv dataset; Figure S16: Top 10 molecular motifs with the highest positive contribution scores on the Lipophilicity dataset; Figure S17: Top 10 molecular motifs with the most negative contribution scores on the Lipophilicity dataset; Table S1: Regression error metrics of MSGRL on the three regression benchmark datasets; Table S2: Statistical comparison between MSGRL and the strongest competing baseline on each of the eight MoleculeNet benchmark datasets; Table S3: Summary of atom features and encoding dimensions used in MSGRL; Table S4: Summary of bond features and encoding dimensions used in MSGRL; Table S5: Summary and statistical overview of the benchmark datasets used for evaluation.
