# Supplementary material for: MSGRL: A Motif-Driven Self-Supervised Graph Representation Learning Framework for Interpretable Molecular Property Prediction
Source: Molecules. 2026 Aug 27;31(17):3008. doi: 10.3390/molecules31173008 (PMC13566960; doi:10.3390/molecules31173008)
Supplement: Supplementary file 1 [file molecules-31-03008-s001.zip › molecules-4489290-supplementary.pdf]

Supplementary materials

MSGRL: A Motif-Driven Self-Supervised Graph Representation Learning Framework for Interpretable Molecular Property Prediction

You Wu, Yuxin Jiang, Xiaoyun Qi, Haitao Fu, Qiyu Tang, Wen Wang*,
Cheng Zeng* and Guosheng Zhu*

Corresponding authors:

Wen Wang, Institute for Neuroscience and Cardiovascular Research, Centre for Cardiovascular Science, University of Edinburgh, Edinburgh EH16 4TJ, UK; Email: wwang5@ed.ac.uk

Cheng Zeng, School of Artificial Intelligence, Hubei University, Wuhan 430062, China, Key Laboratory of Intelligent Sensing System and Security (Hubei University), Ministry of Education, Wuhan 430062, China; Email: zc@hubu.edu.cn

Guosheng Zhu, School of Computer Science, Hubei University, Wuhan 430062, China; Email: zhuguosheng@hubu.edu.cn

CONTENTS

- **Figure S1.** Prediction–ground-truth agreement and distributional comparison of MSGRL on the three regression benchmarks
- **Figure S2.** Top 10 molecular motifs with the highest positive contribution scores on the BACE dataset
- **Figure S3.** Top 10 molecular motifs with the most negative contribution scores on the BACE dataset
- **Figure S4.** Top 10 molecular motifs with the highest positive contribution scores on the BBBP dataset
- **Figure S5.** Top 10 molecular motifs with the most negative contribution scores on the BBBP dataset
- **Figure S6.** Top 10 molecular motifs with the highest positive contribution scores on the SIDER dataset
- **Figure S7.** Top 10 molecular motifs with the most negative contribution scores on the SIDER dataset
- **Figure S8.** Top 10 molecular motifs with the highest positive contribution scores on the ClinTox dataset
- **Figure S9.** Top 10 molecular motifs with the most negative contribution scores on the ClinTox dataset
- **Figure S10.** Top 10 molecular motifs with the highest positive contribution scores on the Tox21 dataset
- **Figure S11.** Top 10 molecular motifs with the most negative contribution scores on the Tox21 dataset
- **Figure S12.** Top 10 molecular motifs with the highest positive contribution scores on the ESOL dataset
- **Figure S13.** Top 10 molecular motifs with the most negative contribution scores on the ESOL dataset
- **Figure S14.** Top 10 molecular motifs with the highest positive contribution scores on the FreeSolv dataset
- **Figure S15.** Top 10 molecular motifs with the most negative contribution scores on the FreeSolv dataset
- **Figure S16.** Top 10 molecular motifs with the highest positive contribution scores on the Lipophilicity dataset
- **Figure S17.** Top 10 molecular motifs with the most negative contribution scores on the Lipophilicity dataset
- **Table S1.** Regression error metrics of MSGRL on the three regression benchmark datasets

- **Table S2.** Statistical comparison between MSGRL and the strongest competing baseline on each of the eight MoleculeNet benchmark datasets
- **Table S3.** Summary of atom features and encoding dimensions used in MSGRL
- **Table S4.** Summary of bond features and encoding dimensions used in MSGRL
- **Table S5.** Summary and statistical overview of the benchmark datasets used for evaluation

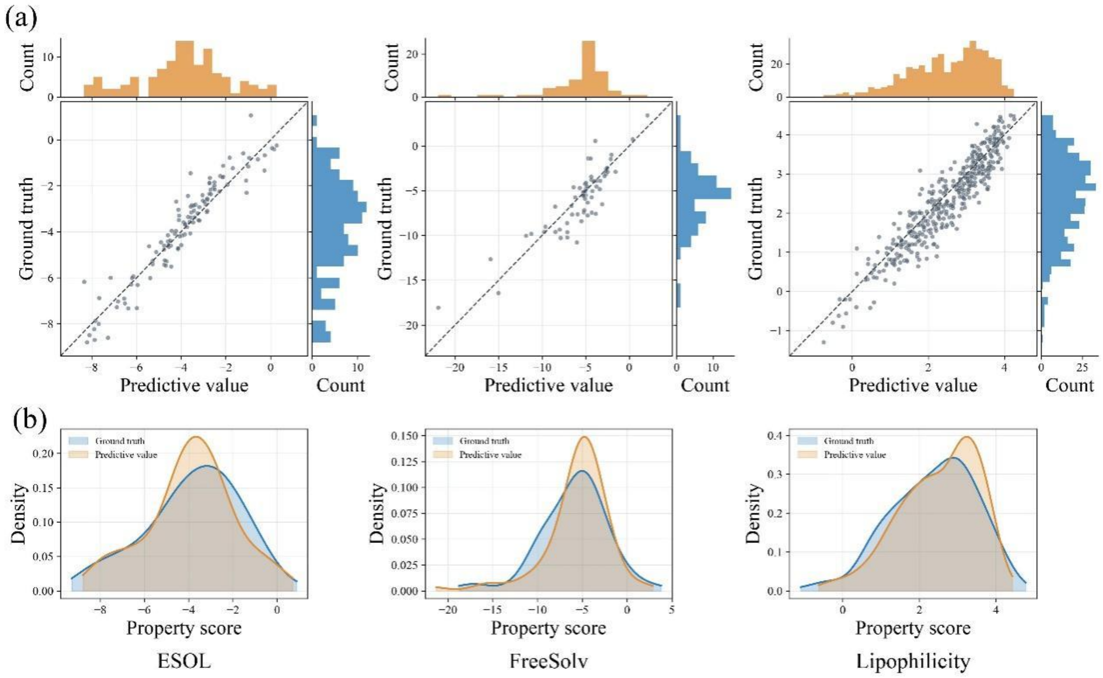

■ Figure S1. Prediction–ground-truth agreement and distributional comparison of MSGRL on the three regression benchmarks. (a) Scatter plots of model predictions versus ground-truth property values for ESOL, FreeSolv, and Lipophilicity, with marginal histograms showing the corresponding distributions. The dashed diagonal line represents ideal agreement between prediction and ground truth. (b) Kernel density estimates comparing the predicted and ground-truth property distributions for the three datasets.

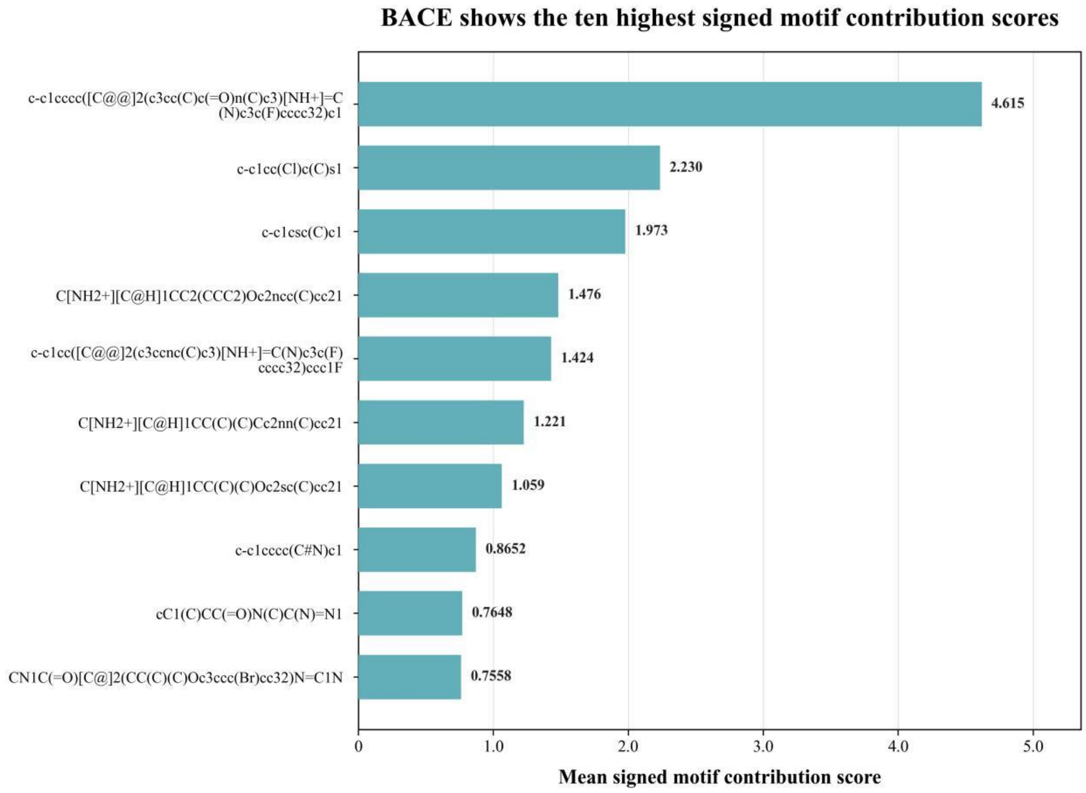

- Figure S2. Top 10 molecular motifs with the highest positive contribution scores on the BACE dataset. The horizontal bar chart ranks motifs based on their mean signed motif contribution scores, where positive values denote key structural fragments that contribute favorably to BACE target activity. Motifs are represented by their corresponding SMILES strings along the vertical axis.

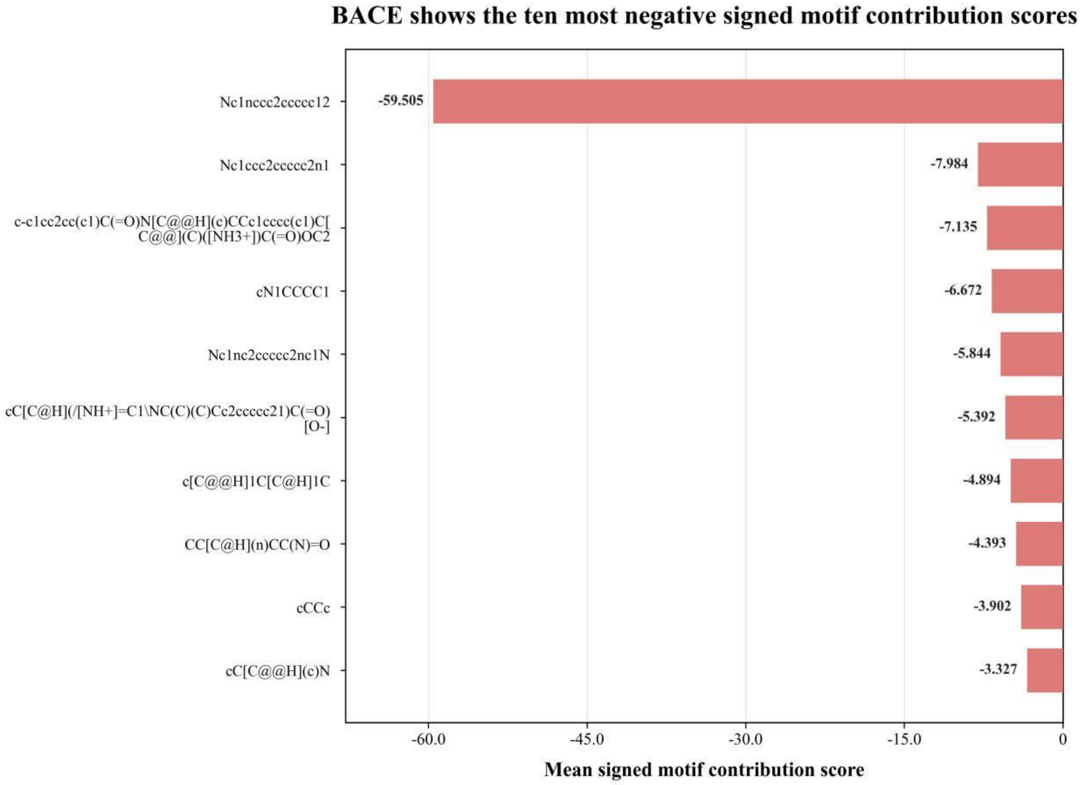

■ Figure S3. Top 10 molecular motifs with the most negative contribution scores on the BACE dataset. The horizontal bar chart ranks motifs based on their mean signed motif contribution scores, where negative values indicate molecular fragments that negatively influence or inhibit BACE activity/target classification. Motifs are represented by their corresponding SMILES strings along the vertical axis.

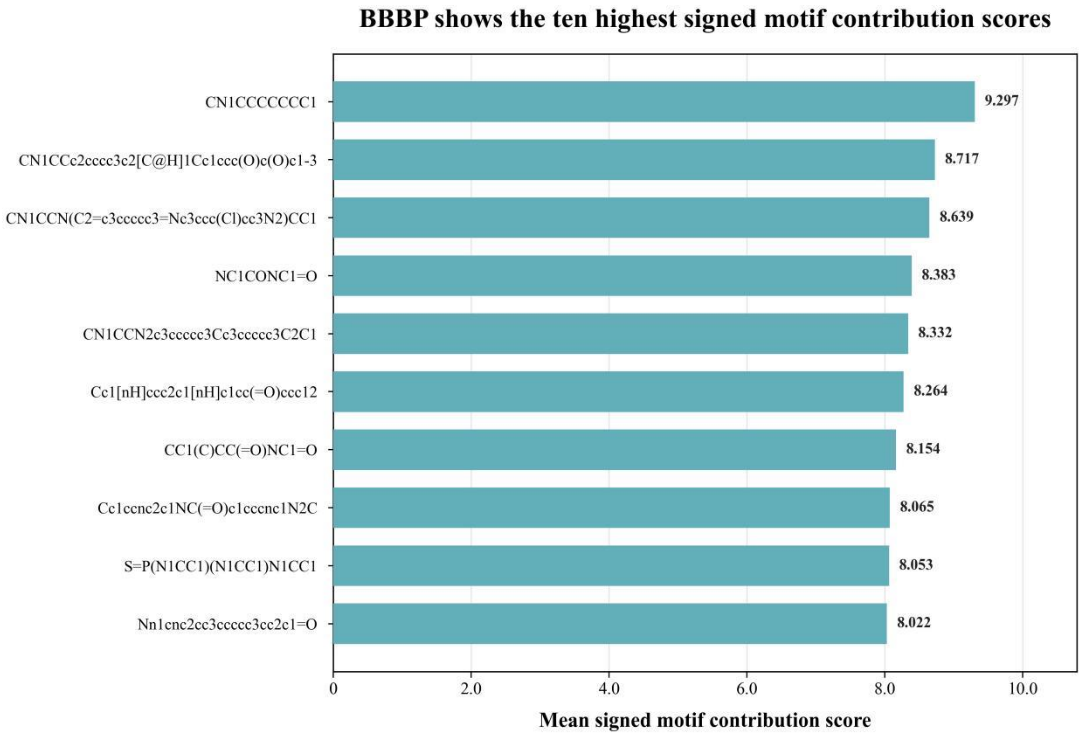

- Figure S4. Top 10 molecular motifs with the highest positive contribution scores on the BBBP dataset. The horizontal bar chart displays motifs ranked by their mean signed motif contribution scores. Positive values indicate motifs associated with increasing the model-predicted probability of blood-brain barrier permeability. Motifs are represented by their corresponding SMILES strings along the vertical axis.

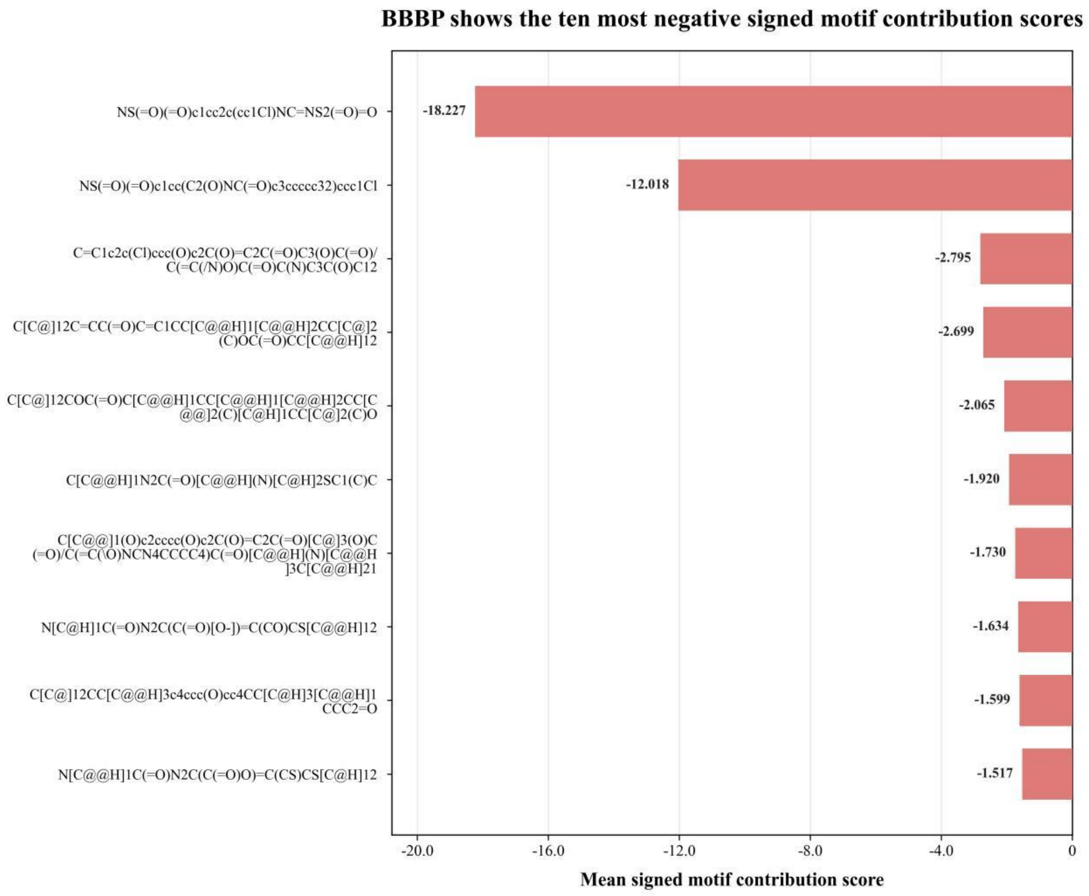

- Figure S5. Top 10 molecular motifs with the most negative contribution scores on the BBBP dataset. The horizontal bar chart displays motifs ranked by their mean signed motif contribution scores, highlighting functional groups and sub-structures that hinder blood-brain barrier permeability. Motifs are represented by their corresponding SMILES strings along the vertical axis.

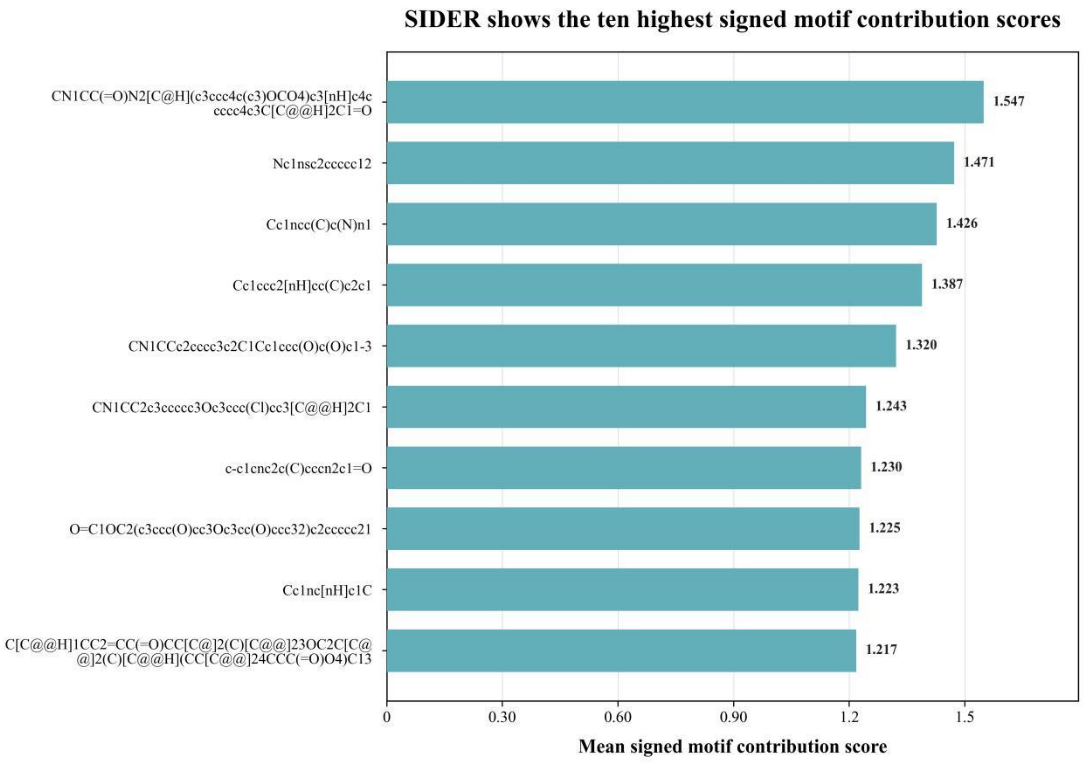

■ Figure S6. Top 10 molecular motifs with the highest positive contribution scores on the SIDER dataset. The horizontal bar chart ranks substructures according to their mean signed motif contribution scores, highlighting key motifs that strongly correlate with or contribute to adverse drug reactions. Motifs are represented by their corresponding SMILES strings along the vertical axis.

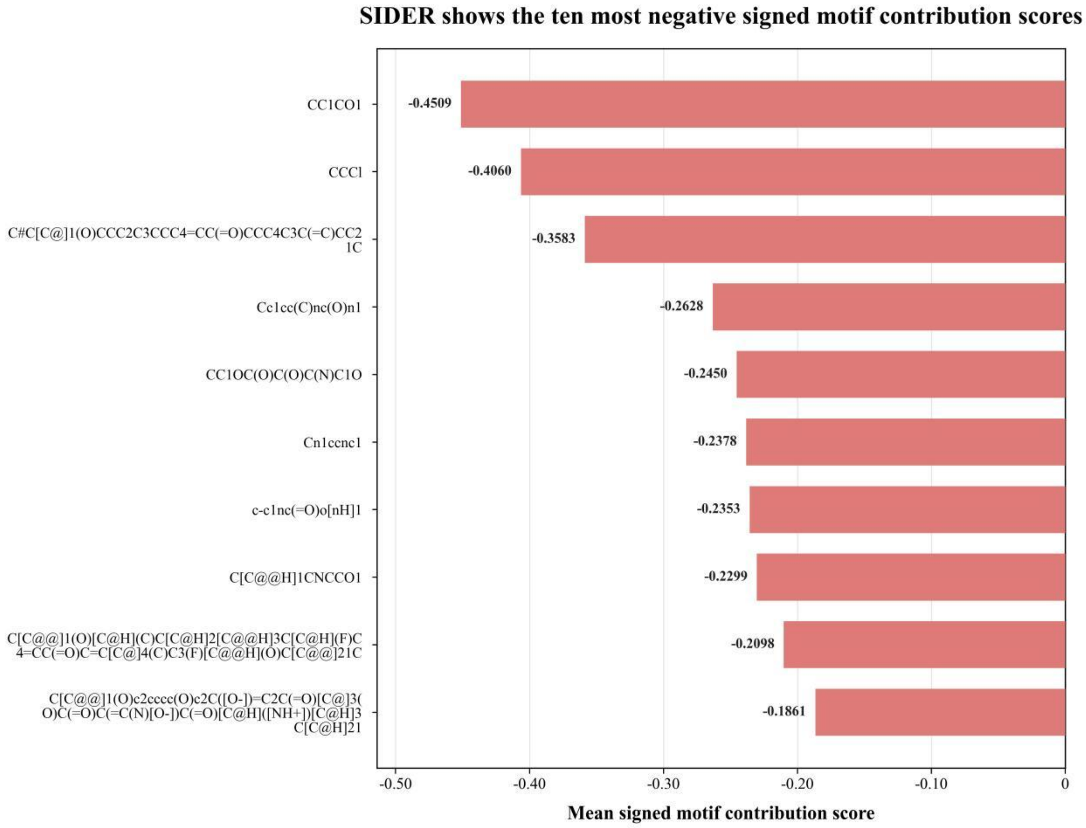

■ Figure S7. Top 10 molecular motifs with the most negative contribution scores on the SIDER dataset. The horizontal bar chart ranks substructures by their mean signed motif contribution scores, where negative values indicate molecular fragments that mitigate or negatively correlate with side-effect occurrences. Motifs are represented by their corresponding SMILES strings along the vertical axis.

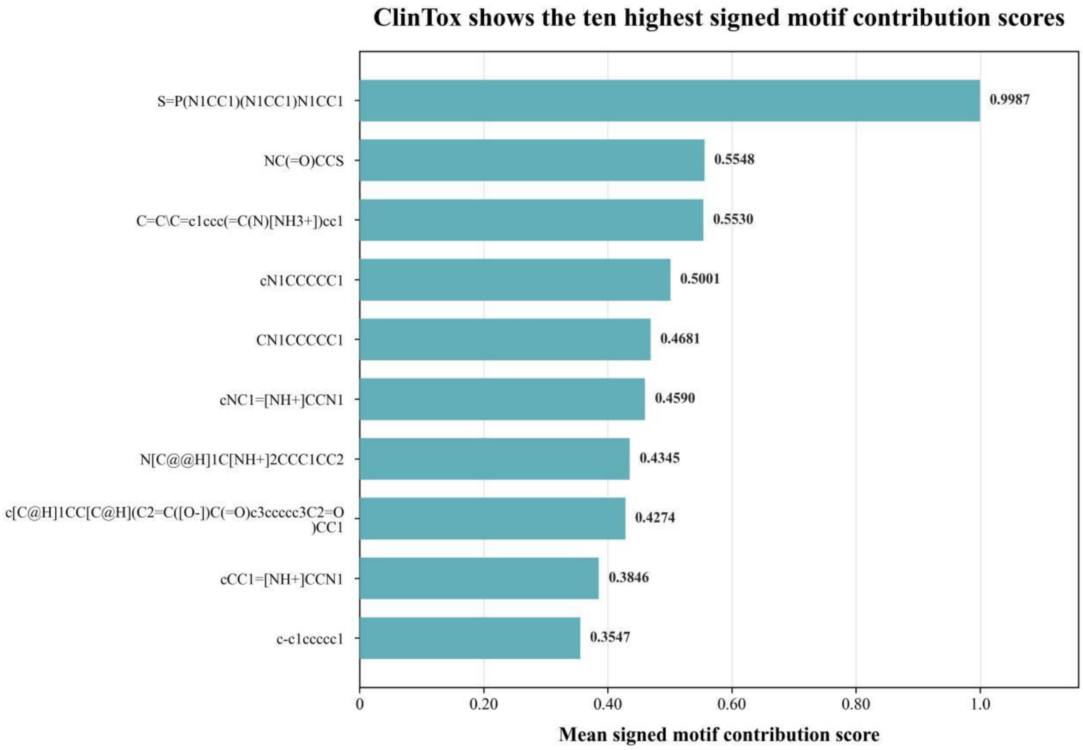

■ Figure S8. Top 10 molecular motifs with the highest positive contribution scores on the ClinTox dataset. The horizontal bar chart ranks motifs based on their mean signed motif contribution scores. Positive values indicate motifs associated with increasing the model output for the positive ClinTox class. Motifs are represented by their corresponding SMILES strings along the vertical axis.

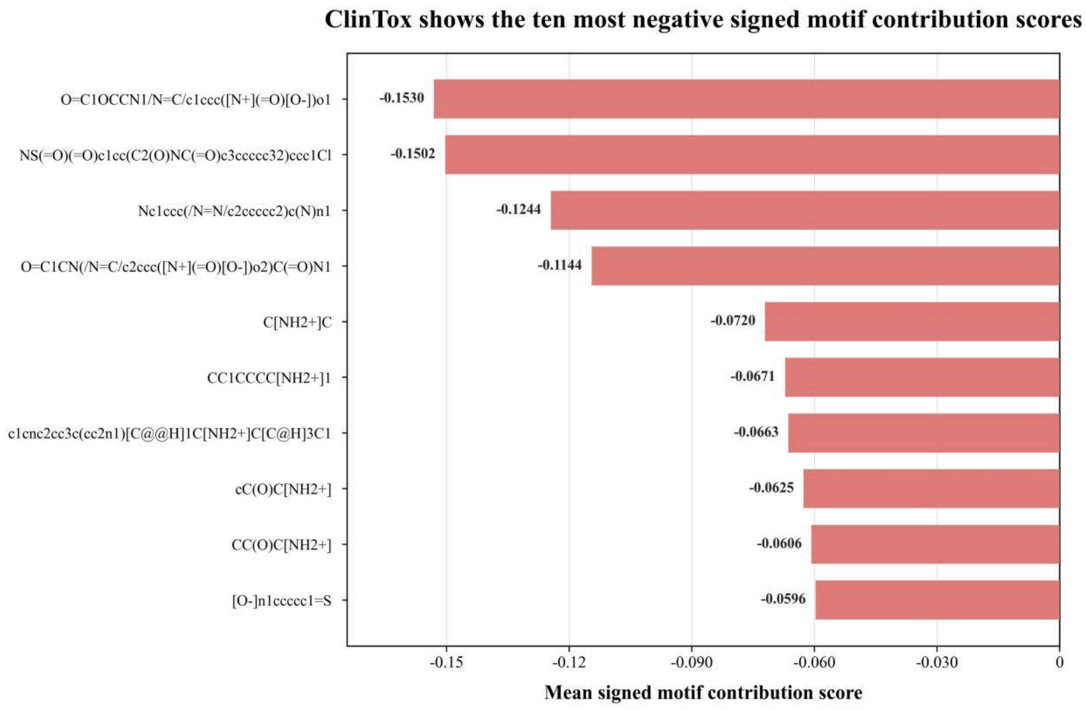

- Figure S9. Top 10 molecular motifs with the most negative contribution scores on the ClinTox dataset. The horizontal bar chart ranks motifs according to their mean signed motif contribution scores. Negative values indicate motifs associated with decreasing the model output for the positive ClinTox class. Motifs are represented by their corresponding SMILES strings along the vertical axis.

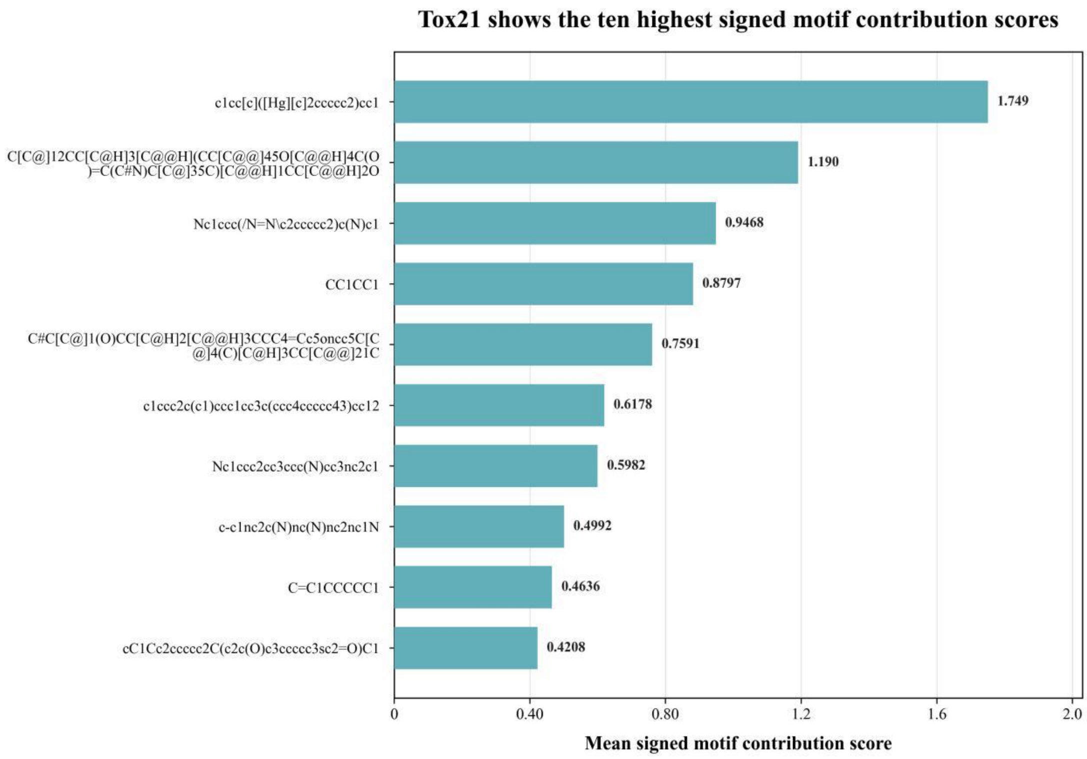

■ Figure S10. Top 10 molecular motifs with the highest positive contribution scores on the Tox21 dataset. The horizontal bar chart ranks motifs with the highest positive mean signed contribution scores for the Tox21 prediction tasks. Motifs are represented by their corresponding SMILES strings along the vertical axis.

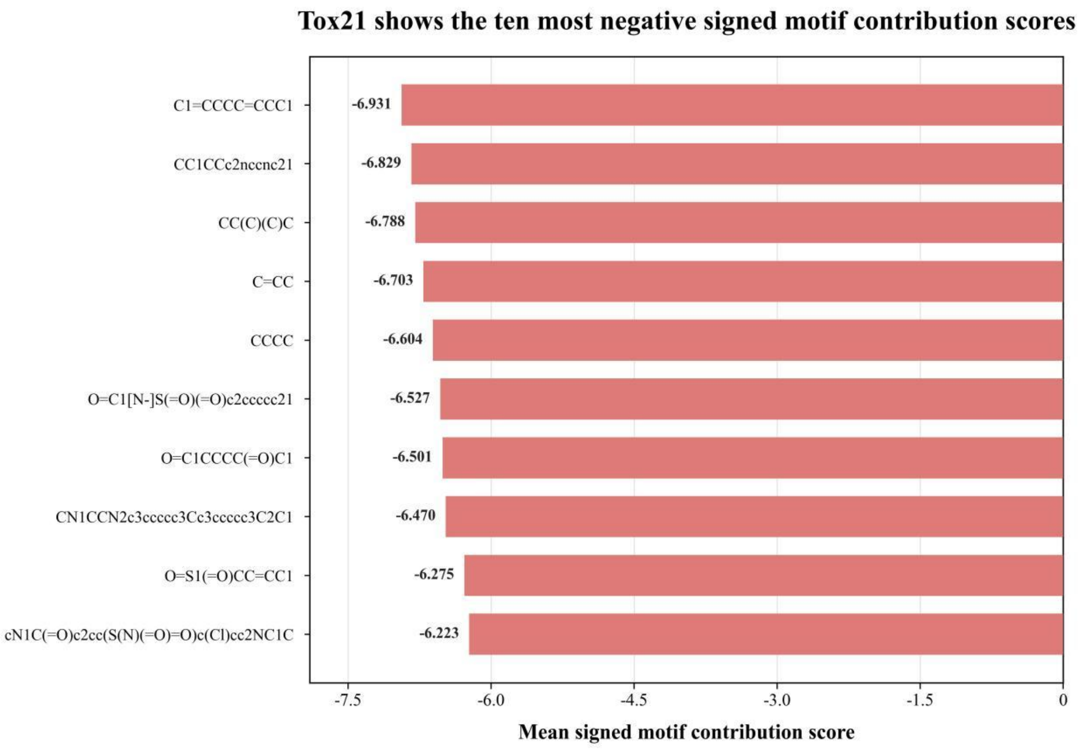

- Figure S11. Top 10 molecular motifs with the most negative contribution scores on the Tox21 dataset. The horizontal bar chart ranks substructures based on their mean signed motif contribution scores, where negative values identify structural fragments that lower biological toxicity signals across assays. Motifs are represented by their corresponding SMILES strings along the vertical axis.

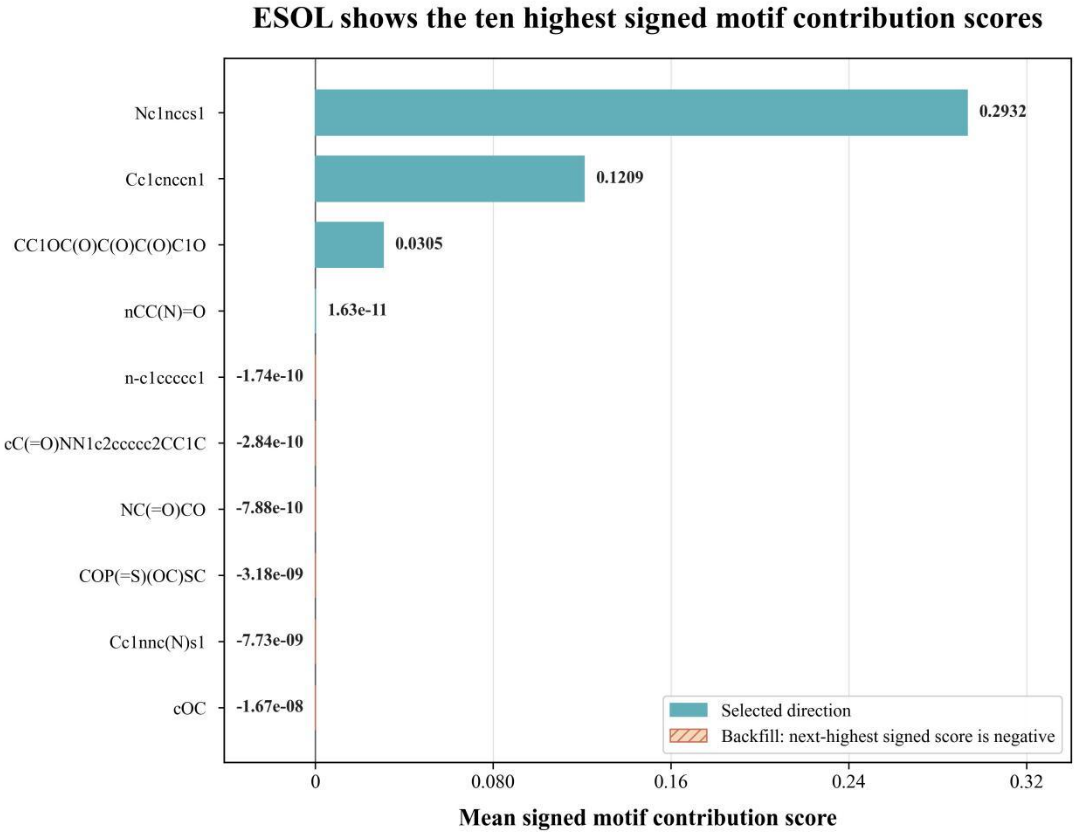

■ Figure S12. Top 10 molecular motifs with the highest positive contribution scores on the ESOL dataset. The horizontal bar chart shows motifs associated with higher predicted ESOL values. The hatched bars (Backfill) represent top ranked entries where the remaining candidate scores become non-positive. Motifs are represented by their corresponding SMILES strings along the vertical axis.

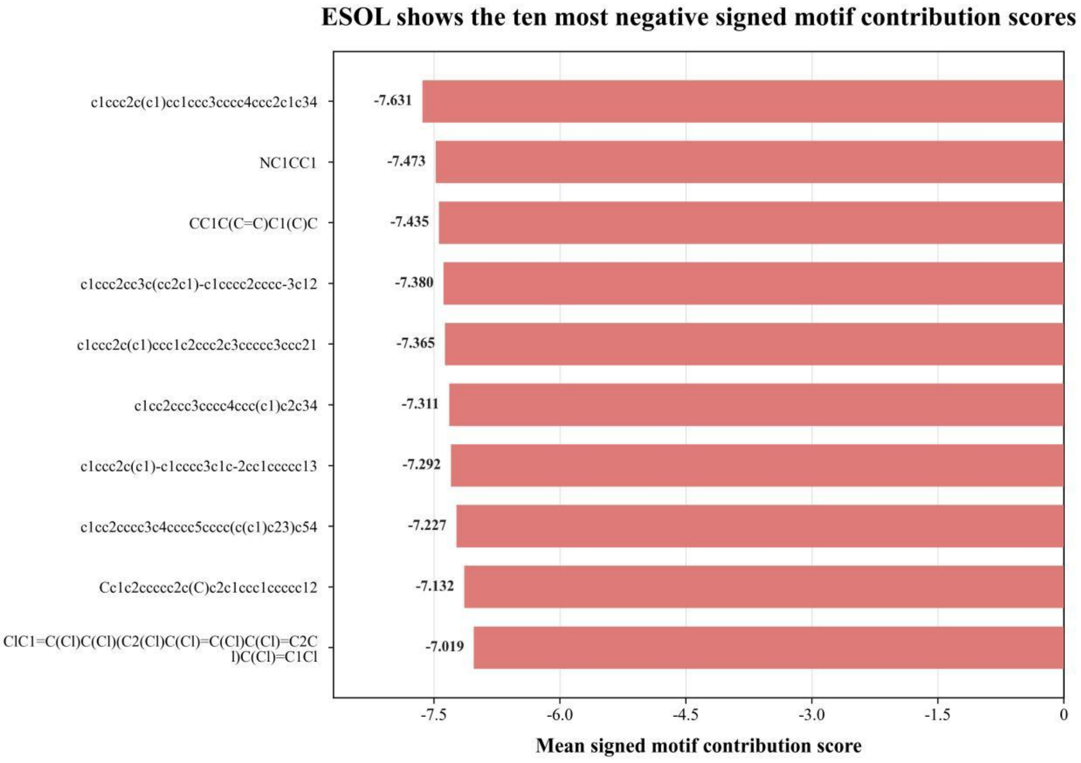

■ Figure S13. Top 10 molecular motifs with the most negative contribution scores on the ESOL dataset. The horizontal bar chart displays motifs ranked by their mean signed motif contribution scores, where negative scores identify motifs with the most negative mean signed contribution scores for ESOL prediction. Motifs are represented by their corresponding SMILES strings along the vertical axis.

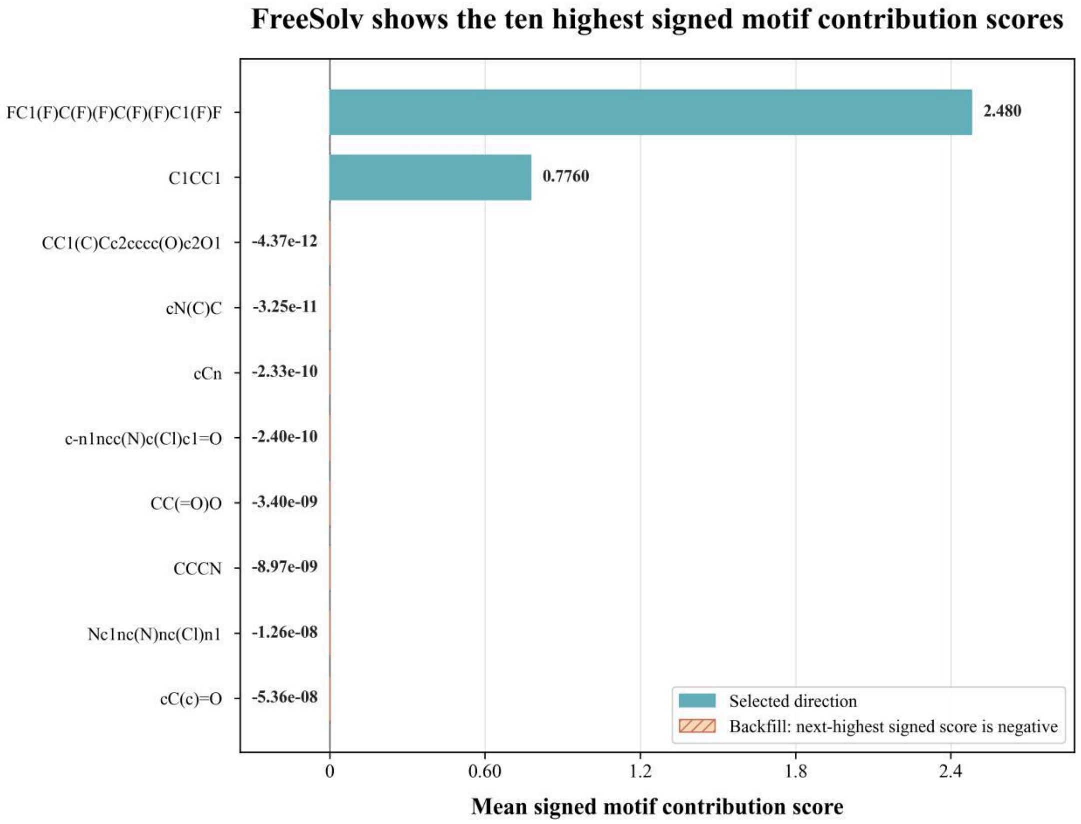

■ Figure S14. Top 10 molecular motifs with the highest positive contribution scores on the FreeSolv dataset. The horizontal bar chart ranks motifs that contribute positively to hydration free energy (disfavoring hydration). The hatched bars (Backfill) indicate candidate motifs backfilled as next-highest values. Motifs are represented by their corresponding SMILES strings along the vertical axis.

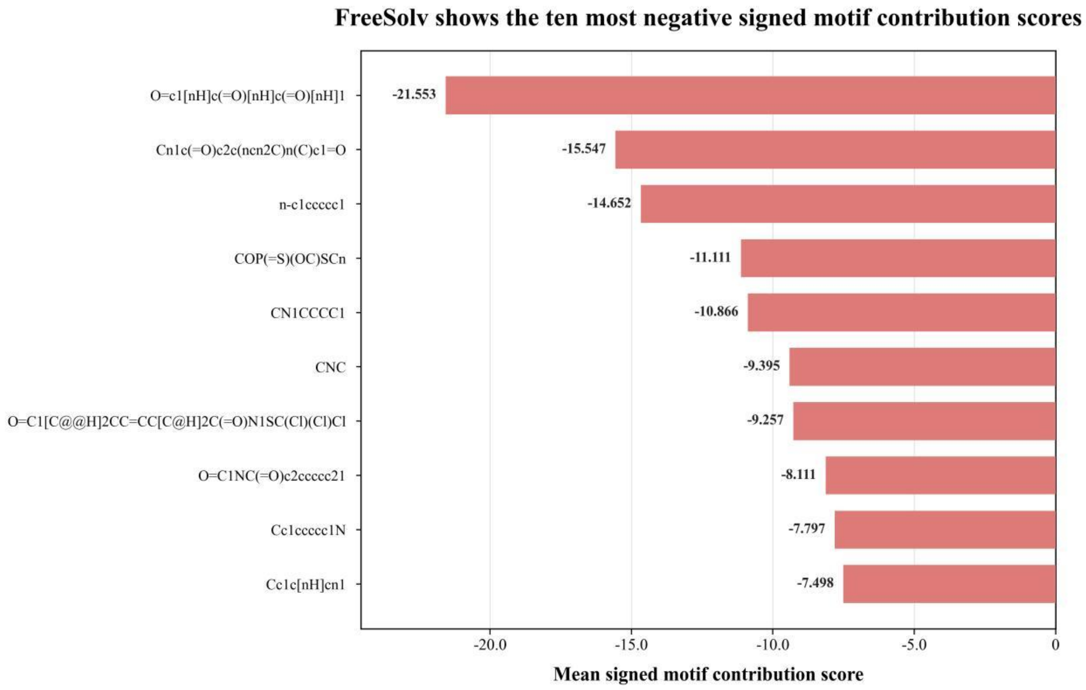

■ Figure S15. Top 10 molecular motifs with the most negative contribution scores on the FreeSolv dataset. The horizontal bar chart ranks motifs by their mean signed motif contribution scores, where negative values indicate polar and hydrogen-bonding substructures that strongly favor hydration (more negative ΔG_{solv}). Motifs are represented by their corresponding SMILES strings along the vertical axis.

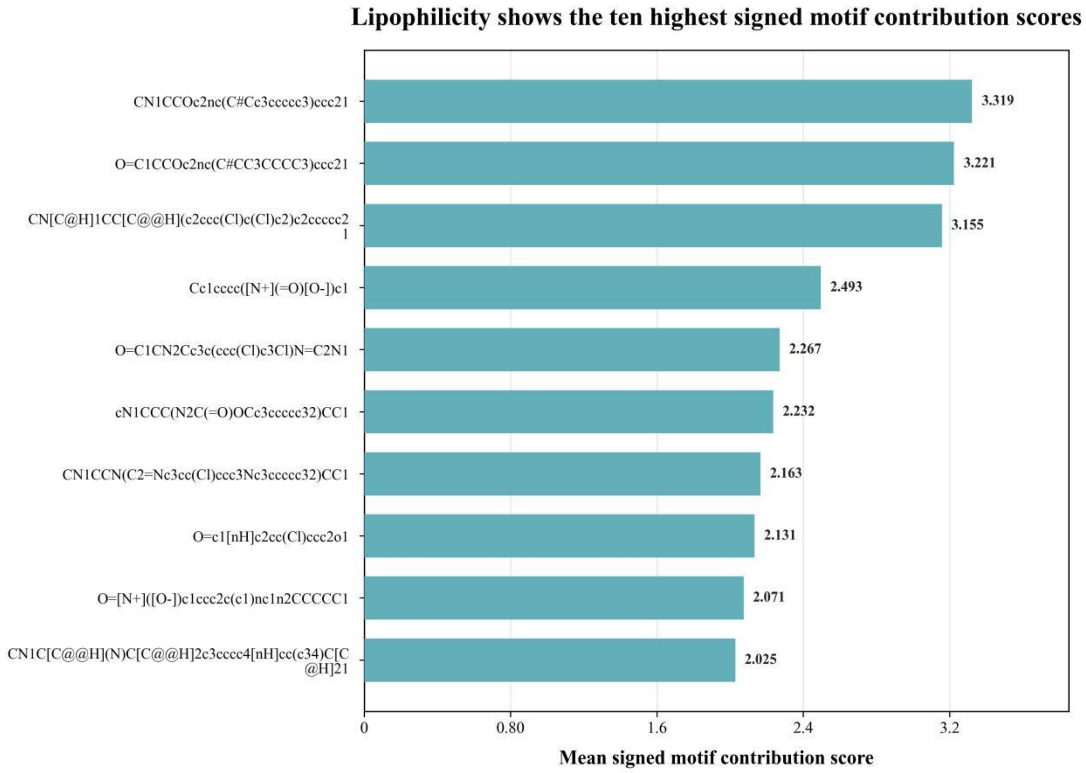

■ Figure S16. Top 10 molecular motifs with the highest positive contribution scores on the Lipophilicity dataset. The horizontal bar chart ranks motifs based on their mean signed motif contribution scores, where positive values denote nonpolar and halogenated/hydrophobic motifs associated with increasing the model-predicted Lipophilicity value. Motifs are represented by their corresponding SMILES strings along the vertical axis.

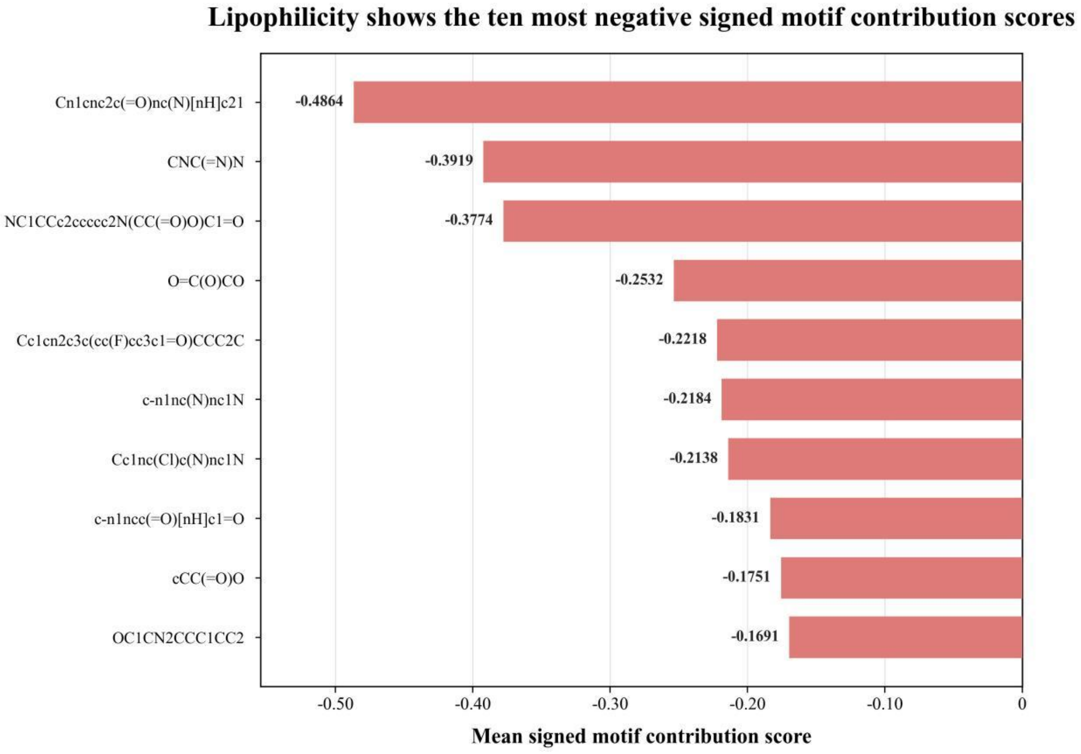

- Figure S17. Top 10 molecular motifs with the most negative contribution scores on the Lipophilicity dataset. The horizontal bar chart ranks motifs based on their mean signed motif contribution scores, where negative values highlight polar and hydrophilic motifs associated with decreasing the model-predicted Lipophilicity value. Motifs are represented by their corresponding SMILES strings along the vertical axis.

- Table S1. Regression error metrics of MSGRL on the three regression benchmark datasets. RMSE values are reported as mean ± standard deviation over ten independent runs. Maximum absolute error values are calculated from the test-set predictions of a single run and therefore represent the largest absolute prediction deviations observed in that run.

**Dataset**	**RMSE**	**Maximum absolute error**
ESOL	0.671±0.003	2.165
FreeSolv	1.939±0.010	4.770
Lipophilicity	0.438±0.021	1.319

■ Table S2. Statistical comparison between MSGRL and the strongest competing baseline on each of the eight MoleculeNet benchmark datasets. Results are reported as mean ± standard deviation over ten runs. Two-sided Welch's t-tests are performed using the ten-run summary statistics, and the resulting p-values are adjusted for multiple comparisons using the Holm procedure.

**Dataset**	**MSGRL**	**Strongest baseline**	**Baseline result**	**Welch's p**	**Holm-adjusted p**
BACE	0.955± 0.010	FraGAT	0.927± 0.002	6.91×10^{-6}	2.07×10^{-5}
BBBP	0.962± 0.012	FraGAT	0.933± 0.006	1.09×10^{-5}	2.18×10^{-5}
SIDER	0.943± 0.018	SCI	0.715± 0.005	1.49×10^{-12}	7.47×10^{-12}
ClinTox	0.972± 0.029	FraGAT	0.969± 0.001	0.751	0.751
Tox21	0.947± 0.006	FraGAT	0.863± 0.005	2.19×10^{-17}	1.54×10^{-16}
ESOL	0.671± 0.003	FraGAT	0.478± 0.012	2.14×10^{-13}	1.28×10^{-12}
FreeSolv	1.939± 0.010	FraGAT	0.538± 0.022	6.43×10^{-23}	5.14×10^{-22}
Lipophilicity	0.438± 0.021	SCI	0.508± 0.011	2.76×10^{-7}	1.10×10^{-6}

- Table S3. Summary of atom features and encoding dimensions used in MSGRL. The table provides detailed specifications of atomic-level features extracted for molecular graph representation in MSGRL. Each atom is characterized by its element type, stereochemical configuration, connectivity degree, formal charge, number of bonded hydrogens, radical count, hybridization state, aromaticity, and ring membership. Categorical features are one-hot encoded with explicit dimensions (Size), with an additional category reserved for unspecified or miscellaneous states.

**Atom feature**	**Description**	**Size**
Atomic number	Atomic number of the element, {1, 2, ..., 118, misc}	119
Chirality type	Chirality type, {CHI_UNSPECIFIED, CHI_TETRAHEDRAL_CW, CHI_TETRAHEDRAL_CCW, CHI_OTHER, misc}	5
Degree of atom	Number of directly bonded atoms, {0, 1, 2, 3, 4, 5, 6, 7, 8, 9, 10, misc}	12
Formal charge	Formal charge of the atom, {-5, -4, -3, -2, -1, 0, 1, 2, 3, 4, 5, misc}	12
Number of hydrogen atoms attached	Number of connected hydrogens, {0, 1, 2, 3, 4, 5, 6, 7, 8, misc}	10
Number of radical electrons	Number of radical electrons, {0, 1, 2, 3, 4, misc}	6
Hybridization type	Hybridization type, {SP, SP2, SP3, SP3D, SP3D2, misc}	6
Aromatic	Whether the atom is part of an aromatic system, {False, True}	2
In ring	Whether the atom is part of a ring system, {False, True}	2

- Table S4. Summary of bond features and encoding dimensions used in MSGRL. The table provides detailed attributes of chemical bond (edge) features utilized in the MSGRL architecture. Features incorporate chemical bond order (single, double, triple, aromatic), stereochemical orientation, and electronic conjugation status. Each attribute is mapped into discrete one-hot vectors corresponding to the specified dimensional sizes.

**Bond feature**	**Description**	**Size**
Bond type	Type of chemical bond, {SINGLE, DOUBLE, TRIPLE, AROMATIC, misc}	5
Bond stereochemistry	The stereo configuration of a bond, {STEREONONE, STEREOZ, STEREOE, STEREOCIS, STEREOTRANS, STEREOANY}	6
Conjugation	whether the bond is conjugated, {False, Ture}	2

- Table S5. Summary and statistical overview of the benchmark datasets used for evaluation. This table gives an overview of the eight public benchmark datasets evaluated in this study, spanning five classification tasks (BACE, BBBP, ClinTox, SIDER, Tox21) and three regression tasks (ESOL, FreeSolv, Lipophilicity). For each dataset, the table reports the number of prediction tasks, task category, primary evaluation metrics (ROC-AUC or RMSE), total number of evaluated molecules, and the total count of extracted sub-structural motifs used during graph modeling.

**Dataset**	**Task**	**Task type**	**Metrics**	**Molecule nums**	**Motif nums**
BACE	1	Classification	ROC-AUC	1513	10930
BBBP	1	Classification	ROC-AUC	2039	8312
Clintox	2	Classification	ROC-AUC	1478	7289
SIDER	27	Classification	ROC-AUC	1427	9510
Tox21	12	Classification	ROC-AUC	7831	27790
ESOL	1	Regression	RMSE	1128	2539
FreeSolv	1	Regression	RMSE	642	1085
Lipophilicity	1	Regression	RMSE	4200	24060
